# Migratory Birds Advance Spring Arrival and Egg‐Laying in the Arctic, Mostly by Travelling Faster

**DOI:** 10.1111/gcb.70158

**Published:** 2025-04-09

**Authors:** Thomas K. Lameris, Michiel P. Boom, Rascha J. M. Nuijten, Nelleke H. Buitendijk, Götz Eichhorn, Bruno J. Ens, Klaus‐Michael Exo, Petr M. Glazov, Sveinn Are Hanssen, Philip Hunke, Henk P. van der Jeugd, Margje E. de Jong, Andrea Kölzsch, Alexander Kondratyev, Helmut Kruckenberg, Olga Kulikova, Hans Linssen, Maarten J. J. E. Loonen, Julia A. Loshchagina, Jesper Madsen, Børge Moe, Sander Moonen, Gerhard J. D. M. Müskens, Bart A. Nolet, Ivan Pokrovsky, Jeroen Reneerkens, Isabella B. R. Scheiber, Hans Schekkerman, Kees H. T. Schreven, Tohar Tal, Ingrid Tulp, Mo A. Verhoeven, Tom S. L. Versluijs, Sergey Volkov, Martin Wikelski, Rob S. A. van Bemmelen

**Affiliations:** ^1^ NIOZ Royal Netherlands Institute for Sea Research Den Burg the Netherlands; ^2^ Groningen Institute for Evolutionary Life Sciences (GELIFES) University of Groningen Groningen the Netherlands; ^3^ Department of Animal Ecology Netherlands Institute of Ecology (NIOO‐KNAW) Wageningen the Netherlands; ^4^ Vogeltrekstation—Dutch Centre for Avian Migration and Demography (NIOO‐KNAW) Wageningen the Netherlands; ^5^ Theoretical and Computational Ecology, Institute for Biodiversity and Ecosystem Dynamics University of Amsterdam Amsterdam the Netherlands; ^6^ Sovon Dutch Centre for Field Ornithology Nijmegen the Netherlands; ^7^ Future For Nature, https://futurefornature.org/ Arnhem the Netherlands; ^8^ Wildlife Ecology & Conservation Group Wageningen University Wageningen the Netherlands; ^9^ Faunabeheereenheid Noord‐Holland Haarlem the Netherlands; ^10^ Michael‐Otto‐Institut im NABU Bergenhusen Germany; ^11^ Institute of Avian Research, Vogelwarte Helgoland Wilhelmshaven Germany; ^12^ Institute of Geography, Russian Academy of Sciences Moscow Russia; ^13^ Norwegian Institute for Nature Research (NINA) Oslo Norway; ^14^ Department of Behavioral and Cognitive Biology University of Vienna Vienna Austria; ^15^ Arctic Centre, University of Groningen Groningen the Netherlands; ^16^ Department of Migration, Max Planck Institute of Animal Behavior Germany; ^17^ Radboud Institute for Biological and Environmental Sciences Radboud University the Netherlands; ^18^ Institute of Biological Problems of the North FEB RAS Magadan Russia; ^19^ Institute for Wetlands and Waterbird Research IWWR e.V Verden (Aller) Germany; ^20^ Department of Ecoscience Aarhus University Aarhus C Denmark; ^21^ Norwegian Institute for Nature Research (NINA) Trondheim Norway; ^22^ Wageningen Environmental Research (WEnR) Wageningen the Netherlands; ^23^ Wageningen Marine Research Wageningen University IJmuiden the Netherlands; ^24^ Institute of Ecology and Evolution, Russian Academy of Sciences Moscow Russia; ^25^ Department of Biology University of Konstanz Konstanz Germany; ^26^ Waardenburg Ecology Culemborg the Netherlands

**Keywords:** Arctic ecosystems, climate change, phenology, seabirds, shorebirds, snowmelt, waterfowl

## Abstract

In the current warming climate, many organisms in seasonal environments advance their timing of reproduction to benefit from resource peaks earlier in spring. For migrants, the potential to advance reproduction may be constrained by their migration strategies, notably their ability to advance arrival at the breeding grounds. Recent studies show various changes in migration strategies, including wintering closer to the breeding grounds, earlier departure from the wintering grounds or faster travels by spending less time at stopover sites. However, whether such changes lead to earlier arrival or earlier breeding remains an open question. We studied changes in migration and reproduction timing in 12 populations of nine migratory birds, including seabirds, shorebirds, birds of prey and waterfowl breeding at Arctic sites bordering the Greenland and Barents Sea, a region undergoing rapid climate warming. The timing of migration and reproduction was derived from tracking and field data and analysed to study (1) how timing has changed in response to the changing moment of snowmelt at the breeding grounds and (2) what adjustments in migration strategies this involved. We found that in years with early snowmelt, egg‐laying in multiple populations advanced, but only two waterfowl populations also advanced arrival in the Arctic. In contrast, arrival in the Arctic generally advanced with time, even when snowmelt or egg‐laying dates did not advance. Earlier arrival with time was mostly explained by populations traveling to the Arctic faster, likely spending less time at stopover sites. Inability to forecast conditions in the Arctic may limit birds to adjust migration timing to annually varying snowmelt, but we show that several species, particularly waterfowl, are able to travel faster and advance the timing of migration over the years. The question remains whether this reflects adaptations to Arctic climate change or other factors, for example, environmental changes along the migratory route.

## Graphical abstract

A warming climate is affecting the phenology of life on earth, including when birds migrate and start breeding. We show that earlier arrival is a general pattern also for birds travelling to the Arctic region, arriving earlier when snow melts early as well as with time. Several species are able to do so by travelling faster, with birds spending less time at stopovers during migration.

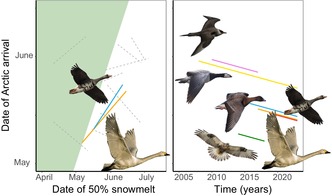

## 1 Introduction

The earth's climate is warming rapidly, resulting in strong environmental changes for all living organisms. One of the most important mechanisms by which climate warming is impacting animal populations, particularly in seasonal environments, is via changes in biotic interactions (Ockendon et al. 2014). Under increasing temperatures, organisms at lower trophic levels, such as plants and arthropods, can strongly advance their phenology (Thackeray et al. 2010). This creates an earlier seasonal ‘resource peak’ for their consumers (Kharouba and Wolkovich 2020) and, as a result, resources may become severely limited late in the season (but see: Reneerkens et al. 2016; Zhemchuzhnikov et al. 2021). Since limited resources during early life can lead to reduced growth or survival of offspring (Boom et al. 2021; Lameris et al. 2018), consumers could be expected to advance their timing of reproduction accordingly. However, large variation exists in the extent to which avian consumers adjust their timing of reproduction to earlier availability of their food (Tavera et al. 2024; Zhemchuzhnikov et al. 2021). Some species appear able to synchronise reproduction with food availability (Rakhimberdiev et al. 2018), while others show little or even no adjustment in timing (Keogan et al. 2018; Lameris et al. 2022; Reneerkens et al. 2016). For migratory species, advancements in the timing of reproduction are often considered to be constrained by the timing of migration (Both 2010; Both and Visser 2001), yet there are several examples of migratory species for which laying dates are to some extent independent of arrival dates (Lameris et al. 2018; Lourenço et al. 2011). For many species, it thus remains an open question as to whether advancements in laying dates are limited by the potential for earlier migratory arrival on the breeding grounds.

In theory, earlier migratory arrival is achievable by three, not mutually exclusive, mechanisms: (1) shortening migration distances by spending the non‐breeding period closer to the breeding grounds (Visser et al. 2009), (2) earlier departure from the non‐breeding grounds (Conklin et al. 2021) and (3) faster travel between non‐breeding and breeding sites (Lameris et al. 2018; Morbey and Hedenström 2020). These strategies require responses at different instances during the migratory journey and may be limited by different constraints.

The location where migrants choose to spend the non‐breeding period generally depends on local weather conditions and food availability, which ensure survival. As such, the first mechanism (spending the non‐breeding period closer to the breeding grounds) is often associated with temperature increase (Linssen et al. 2023; Maclean et al. 2008; Visser et al. 2009) and increases in food availability (Clausen et al. 2018), enabling birds to survive in non‐breeding areas closer to the breeding grounds. It has been hypothesised that from these areas, birds may be better able to predict the onset of spring in breeding sites, enabling earlier arrival (Visser et al. 2009).

The second mechanism, earlier departures from non‐breeding grounds, is generally considered a response to changing environmental conditions, either at non‐breeding grounds or at stopover sites further along the flyway (Conklin et al. 2021; Lisovski et al. 2024), and therefore is limited by the extent to which changing conditions are known from experience or can be predicted from afar (Kölzsch et al. 2015; Lameris, Scholten, et al., 2017; Tombre et al. 2008). Earlier departure will also be constrained by the ability to build up sufficient energy stores prior to departure, which need to be built up faster (Lindström et al. 2019) or initiated earlier (Ouwehand et al. 2023), as well as by the completion of plumage moult in some species (Newton 2009). Hence, earlier departures will importantly depend on local benign conditions for fuelling and moulting in the non‐breeding area.

The third mechanism, increasing travel speed between non‐breeding and breeding sites, can be achieved by spending less time at stopover sites and is probably a response to changing conditions *en route* (Lameris et al. 2018). Species can increase their travel speed by spending less time on stopover sites (Lameris et al. 2018; Rakhimberdiev et al. 2018), achieved either by building up energy stores at stopover sites faster (Lindström et al. 2019) or by departing with fewer stores, leading to arrival on their breeding site with a lower body mass (Lameris et al. 2018). Therefore, earlier departure from non‐breeding sites or higher travel speed by spending less time on stopovers represent different choices on where to fuel body stores: in non‐breeding sites before departure, on stopover sites during migration (Evans and Bearhop 2022) or at the breeding site after migration to recover before reproduction (Lameris et al. 2018). While those choices may result in earlier arrival timing, they may not necessarily allow for earlier breeding as this will also depend on energy stores (Bêty et al. 2003).

Whether birds can advance spring migration timing by shortening migration distances, earlier departures or faster travels will be linked to the environmental and internal constraints they face, which may vary among populations. For example, shifts in the non‐breeding range may only be possible following changes in environmental conditions in the non‐breeding range (Nuijten et al. 2020); advancements in the timing of departure may only be possible when conditions for fuelling at non‐breeding sites improve (Ouwehand et al. 2023), as well as conditions on subsequent stopover sites; and an increase in migration speed by decreasing stopover time can likely only be achieved by birds fuelling faster on stopovers or travelling with less internal energy stores. To better understand what limits the advancement of migratory arrival and egg‐laying, we here study changes in migration and reproduction timing in a range of arctic‐breeding migratory bird populations. These populations collectively face warming conditions on their breeding grounds yet differ in migration strategy and likely vary in environmental and internal constraints during migration.

Arctic migratory birds form an ideal system to study variation in migratory advancements. The Arctic is a highly seasonal environment, which is warming four times faster than elsewhere (Rantanen et al. 2022). As such, the effects of warming are more easily detectable and are expected to have a large impact on species breeding here. The Arctic is home to many migratory breeding birds, ranging from small passerines to large waterfowl, with migration distances ranging between 1,400 (e.g., rough‐legged buzzard *Buteo lagopus* , Pokrovsky et al. 2024) and 25,000 km (Arctic tern *Sterna paradisaea* , Egevang et al. 2010). Arctic climate warming is resulting in earlier snowmelt in spring (Box et al. 2019) which is a major determinant of the time suitable for the reproduction of migratory birds. Snowmelt both frees up available nesting sites and triggers forage plant growth (Cooper et al. 2011; Semenchuk et al. 2016) and arthropod emergence (Chagnon‐Lafortune et al. 2024; Leingärtner et al. 2014), which are key resources for herbivorous and insectivorous birds. Earlier snowmelt is therefore expected to have major effects on the phenology of resources for birds (Lameris, Jochems, et al., 2017; Shaftel et al. 2021; Tulp and Schekkerman 2008). Facing rapid environmental changes, arctic migratory birds would be predicted to make similarly rapid adjustments in the timing of migration and reproduction in response. However, not all species seem to do so (Keogan et al. 2018; Post et al. 2018; Zhemchuzhnikov et al. 2021). Therefore, it is likely that some species are encountering constraints that limit their ability to advance migration timing.

We investigate the annual and long‐term variation in the timing of migratory arrival and egg‐laying on the breeding grounds in response to local changes in snowmelt conditions in a suite of nine migratory bird species breeding in the Barents and Greenland Sea region. We expect changes in timing with earlier snowmelt to vary between populations due to varying constraints, with the largest flexibility in timing for larger species of waterfowl, which bring additional 'capital' energy stores on migration (Klaassen et al. 2006) and have already shifted their non‐breeding grounds further northward (Nuijten et al. 2020). Over time, we expect changes in migration timing to follow changes in snowmelt. We further analyse the mechanisms behind the variations and trends in arrival, examining whether these are explained by shifts in non‐breeding ranges, departure timing, or travel speed. Finally, we consider whether the timing of arrival potentially limits advancements in the timing of egg laying. The variation among species and populations is discussed in the light of environmental and internal constraints, capitalising on differences in migration and reproduction strategies among species and populations, as well as on other existing case studies with approaches similar to ours.

## 2 Methods

We gathered data on the timing of spring migration and the timing of egg‐laying in relation to the local timing of snowmelt for a suite of arctic‐breeding bird species, using a large set of tracking data supplemented by field data on egg‐laying dates. Rather than using tracking data of individuals tracked from the non‐breeding grounds and breeding across the entire breeding range of a species, we focussed on tracking data of individuals breeding at specific Arctic breeding sites. In this way, we aimed to reduce variation in the timing of arrival in the Arctic caused by variations in breeding location (e.g., Conklin et al. 2010). All data and scripts for analyses can be found in Lameris et al. (2025).

### 2.1 Study Species and Sites

**FIGURE 1:**
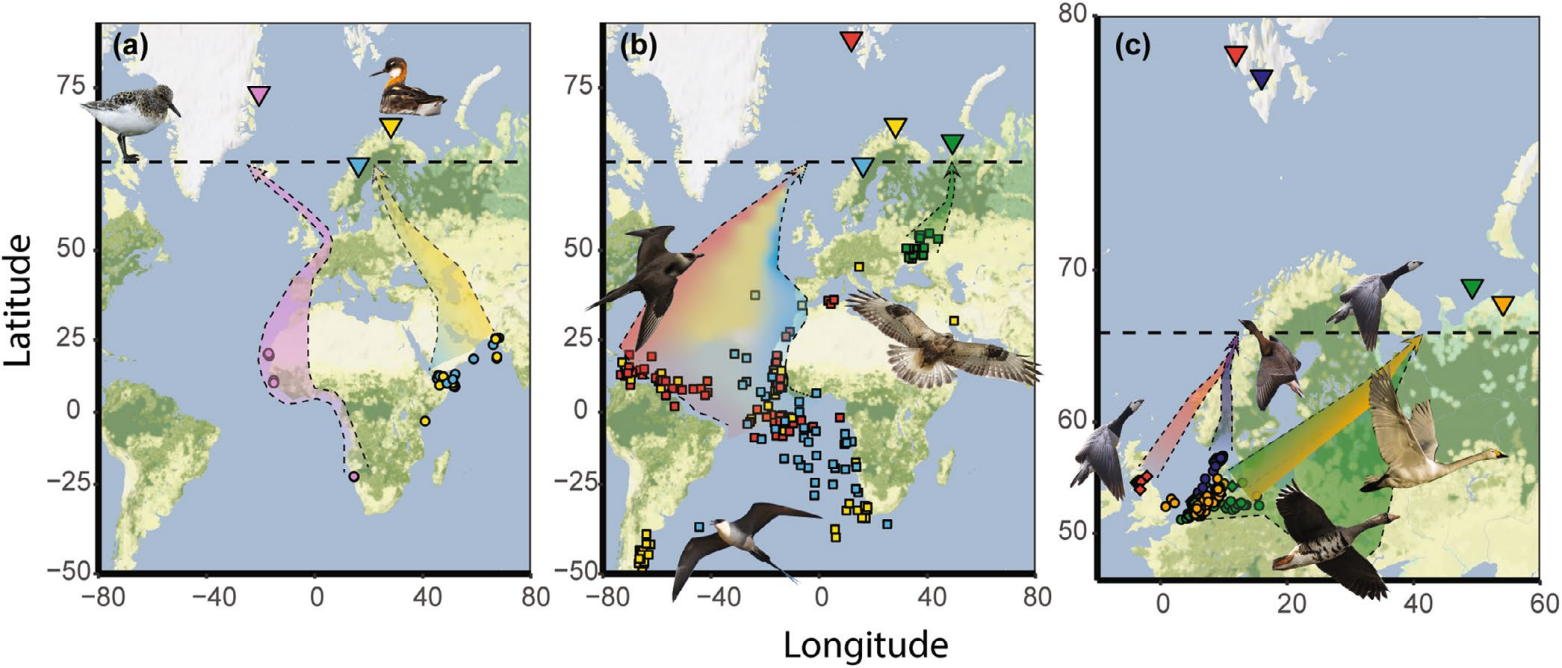
Wintering centroids (median positions) of individual birds per year, general migration routes and study sites in the Arctic. Dots, squares and diamonds show wintering centroids and inverted triangles show study sites, with in (a) sanderlings breeding at Zackenberg (pink) and red‐necked phalaropes breeding at Ammarnäs (light blue) and Slettnes (yellow), (b) Arctic skuas (dots) breeding at Kongsfjorden (red) and Slettnes (yellow), long‐tailed skuas (squares) breeding at Ammarnäs (light blue) and rough‐legged buzzards breeding at Kolguev Island (green dots), and (c) barnacle geese (green diamonds) and greater white‐fronted geese (green dots) breeding on Kolguev Island, barnacle geese breeding in Kongsfjorden (red diamonds), pink‐footed geese breeding in Adventdalen (dark blue dots) and tundra swans breeding at the Malozemelskaya tundra (orange dots). Broad migration routes are indicated with dotted lines and colours similar to the study sites and wintering centroids. In (c), barnacle geese breeding on Kolguev Island follow the northern green route while greater white‐fronted geese use the entire green route. The dotted line indicates the Arctic circle. Photos by Tim Sudlow (tundra swan), Nick Goodrum (pink‐footed goose), Jasper Koster (barnacle goose), Nick Athanas (sanderling, greater white‐fronted goose), John Quine (Arctic skua), Matti Suopajärvi (rough‐legged buzzard) and Alaska Region U.S. Fish & Wildlife Service (red‐necked phalarope, long‐tailed skua). Map tiles by Stamen Design, under CC BY 4.0. Data by OpenStreetMap, under ODbL.

Our dataset contained tracking data (519 spring migrations of 274 individual birds) from 12 populations and nine migratory bird species, breeding at seven Arctic study sites in the Barents and Greenland Sea region (Figure 1; Table 1) from the period 2007–2023. For this paper, we treat each species at a separate study site as a separate population. The species and study sites included greater white‐fronted goose *Anser albifrons* (Kolguev Island, north‐western Russia), pink‐footed goose *Anser brachyrhynchus* (Adventdalen, Svalbard), barnacle goose *Branta leucopsis* (Kolguev Island, north‐western Russia and Kongsfjorden, Svalbard), tundra swan *Cygnus colombianus* (Malozemelskaya tundra, north‐western Russia), red‐necked phalarope *Phalaropus lobatus* (Ammarnäs, northern Sweden and Slettnes, northern Norway), sanderling *Calidris alba* (Zackenberg, north‐eastern Greenland), Arctic skua *Stercorarius parasiticus* (Slettnes, northern Norway and Kongsfjorden, Svalbard), long‐tailed skua *Stercorarius longicaudus* (Ammarnäs, northern Sweden) and rough‐legged buzzard (Kolguev Island, north‐western Russia). Birds were tracked using GPS loggers, GPS‐GSM‐transmitters, satellite transmitters (GPS‐PTT) or geolocators (Global Location Sensing, GLS, Table 1). We tracked only females of the three goose species and both females and males of the other species. Most birds were captured and fitted with tracking devices on their breeding grounds (Table 1), and the study sites of these populations, defined as the area in which birds were captured, were relatively small (8–56 km^2^, Table S1). Only greater white‐fronted geese, barnacle geese (north‐western Russian population) and tundra swans were captured and tracked from their non‐breeding grounds in the Netherlands and Germany. For these populations, we selected larger areas as study sites (6,061–15,132 km^2^) known to host major concentrations of these species (Glazov et al. 2021; Kondratyev et al. 2013; Rees 2010), namely Kolguev Island (greater white‐fronted geese, barnacle geese) and the Malozemelskaya tundra region, west of the Pechora Sea (tundra swans). Only individuals who initiated a nest in these study sites were included (see methods section ‘Date of egg laying’). For tundra swans, nesting behaviour was more difficult to detect from tracking data, and we developed a more comprehensive method to evaluate this making additional use of acceleration data (supplemental materials). To increase the sample size for this species, we also included male individual swans that stayed in the Malozemelskaya tundra study site for the entire month of June. Rough‐legged buzzards were captured and tracked from various locations on Kolguev Island (Pokrovsky et al. 2024), and we therefore used the study area of Kolguev Island also for this population. Detailed information on tracking methods can be found in the species‐specific studies (for references and sample sizes, see Table 1).

**TABLE 1:** Study populations, including information on the number of spring migration tracks and dates of egg laying of tracked birds, the number of additional egg laying dates, details on the type of tracking device used, study ID on Movebank and the reference in which details on the study can be found.

Species	Breeding location of population	Study years	# tracks with arrival in Arctic (# individuals)	# laying dates from tracked birds	# laying dates from other birds	Tracking device type	Movebank ID's	References
*Barnacle goose*	Svalbard (Kongsfjorden)	2021–2023	33 (19)	33 (19)	—	GPS‐GSM	1,176,499,696	de Jong et al. (2024)
	North‐western Russia (Kolguev Island)	2008–2011, 2017–2023	84 (49)	84 (49)	—	Satellite transmitter, GPS‐GSM	29,799,425, 1,114,583,459, 137,654,491	van der Jeugd et al. (2014) and Boom, Lameris, et al. (2023)
*Greater white‐fronted goose*	North‐western Russia (Kolguev Island)	2014, 2016 – 2023	38 (12)	38 (12)	—	GPS‐logger, GPS‐GSM	12,135,743, 180,156,318, 9,589,196, 44,083,081	van Wijk et al. (2012) and Kölzsch et al. (2016)
*Pink‐footed goose*	Svalbard (Adventdalen)	2019–2023	26 (11)	26 (11)	—	GPS‐GSM	1,239,582,803	Schreven et al. 2021
*Tundra swan*	North‐western Russia (Malozemelskaya tundra)	2017–2023	41 (23)	12 (9)	—	GPS‐GSM	181,958,207, 1,881,288,761, 1,812,016,959	Nuijten and Nolet (2020) and Linssen et al. (2023)
*Red‐necked phalarope*	Central Sweden (Ammarnäs)	2014–2017	12 (11)	—	65	Geolocator	99,570,338	van Bemmelen et al. (2019)
	Northern Norway (Slettnes)	2015–2017	10 (7)	—	83	Geolocator	968,983,708	van Bemmelen et al. (2019)
*Sanderling*	Northeast Greenland (Zackenberg)	2014, 2016, 2017	9 (8)	5	92	Geolocator		Reneerkens et al. (2019 )
*Arctic skua*	Svalbard (Kongsfjorden)	2010–2017	71 (35)	67 (34)	—	Geolocator	2,746,281,685	van Bemmelen et al. (2024)
	Northern Norway (Slettnes)	2015–2018	60 (41)	53 (36)	—	Geolocator	968,984,187	van Bemmelen et al. (2024)
*Long‐tailed skua*	Central Sweden (Ammarnäs)	2012–2015	38 (21)	—	89	Geolocator	968,980,842	van Bemmelen et al. 2017
*Rough‐legged buzzard*	North‐western Russia (Kolguev Island)	2014–2017	14 (7)	7	—	GPS‐GSM	9,493,874	Pokrovsky et al. (2021)

### 2.2 Tracking Data Preprocessing

Tracking data were acquired from Movebank (Kays et al. 2022) and datasets from authors, with all data sources described in Table 1. For Arctic skuas, long‐tailed skuas, red‐necked phalaropes and sanderlings, we used previously obtained tracks from GLS data as described in (van Bemmelen et al. 2017, 2019, 2024; Reneerkens et al. 2019). All studies used a light intensity threshold of 2.0 to determine twilight events. To avoid large errors around the equinox, latitude data were deleted from 14 days prior to 18 days after the spring equinox. For greater white‐fronted geese (Kölzsch et al. 2016), barnacle geese (Boom, Lameris, et al., 2023; Kölzsch et al. 2015), pink‐footed geese (Schreven et al. 2021), tundra swans (Linssen et al. 2023; Nuijten and Nolet 2020) and rough‐legged buzzards (Pokrovsky et al. 2021), previously published GPS tracks were downloaded from Movebank. GPS tracks were filtered to exclude outliers, identified as positions where the speed required to travel from that to the next position was larger than 120 km/h [which is 10 km/h faster than recorded flight speeds for these species, (Alerstam et al. 2007; Miller et al. 2005)]. We only included GPS‐ and geolocator tracks that contained at least one position per day from January until arrival at the Arctic Circle.

### 2.3 Migration Timing, Migration Distance and Travel Speed

From tracking data, we extracted individual data on spring migration timing including departure from the non‐breeding area and arrival in the breeding area, as well as total migration distance and travel speed. First, individual non‐breeding sites were calculated as mid‐winter geographical centroids: the median position during January and February, a period during which all populations are considered to be present in their non‐breeding regions. The calculation of the departure date from the non‐breeding site differed between geolocation‐ and GPS‐based tracks. For GPS tracks, we defined the departure date as the date with the last position in spring within 200 km of the mid‐winter centroid. For GLS tracks, which suffer from a larger positional error, especially in latitude during the period around the equinox (Lisovski et al. 2012), we closely inspected raw position estimates to find the first date with consistent directional movement away from the non‐breeding site.

The arrival date in the Arctic was determined as the date at which birds crossed the Arctic Circle (latitude 66.33° N). For GPS data, this was defined as the first date above 66.33° N. GLS tracks are calculated based on sunrise and sunset events and therefore cannot be calculated for positions above the Arctic Circle during the polar day with 24 h of continuous light (Lisovski 2018). For these tracks, we defined crossing of the Arctic Circle as the first day after the date with the last dark night (which usually occurred at c. 60° N latitude). An earlier study found that the crossing from these last positions to the Arctic is generally rapid without stops (van Bemmelen et al. 2024).

Total migration distance was calculated as the cumulative sum of great circle distances between daily averaged positions, from departure from the non‐breeding site to arrival in the Arctic (at the Arctic Circle). Travel speed was calculated as the total migration distance divided by the time between departure and arrival in the Arctic in km/day. Due to high flight speeds, the actual time spent flying is relatively short; therefore, variation in migration speed mostly represents variation in the amount of time spent on stopover sites along the migratory routes (Alerstam and Bäckman 2018). We emphasise that our measure of travel speed is not the same as migration speed, which includes the time spent fuelling at non‐breeding sites before departure (Alerstam 2003).

### 2.4 Date of Egg‐Laying

The date of egg‐laying, defined as the date at which the first egg was laid, was determined from (i) field observations, (ii) patterns in light measurements, and (iii) GPS tracking data. (i) In field observations, the date of egg‐laying for individuals with and without tracking devices was either observed directly, back‐calculated from incomplete clutches in geese (van der Jeugd et al. 2009) and shorebirds (Liebezeit et al. 2014; Reneerkens et al. 2016), back‐calculated from observed hatching dates [by subtracting the period required for incubation, based on (Cramp and Perrins 1988) or assessed by egg floatation (Liebezeit et al. 2007)]. (ii) For GLS data, laying dates were determined based on regular periods of darkness of at least 1 h while the bird was in an area with continuous daylight (Verhoeven et al. 2020). (iii) For GPS data of geese (of which only females were tagged), the timing and position of nesting were determined from GPS locations after arrival on the breeding site, where the nest location was determined as a position where the daily standard deviation in latitude was less than 25.4 m (Schreven et al. 2021) for at least three consecutive days. The first of these 3 days was then determined as the date of egg‐laying. For greater white‐fronted geese and barnacle geese from Kolguev Island, for which both population‐average laying dates from field data as well as extracted from tracking data were available, yearly averaged laying dates correlated strongly between both methods (Pearson's correlation = 0.83, *t* = 4.38, *p* = 0.002, *n* = 9 years). In contrast to geese, where only the female incubates the eggs, in tundra swans, females and males take turns to sit on the nest. This makes it more difficult to distinguish nest positions, and we therefore used a modified method for which also accelerometer data were used (for details see Supporting Information Methods). For GPS data of rough‐legged buzzards, we determined the start of nesting as the first day in a period for which a bird stayed within one position, that is, a difference between GPS positions of less than 3 m, for more than 1 day. This method was verified using accelerometer data (for details, see Curk et al. 2022). For long‐tailed skuas, red‐necked phalaropes and sanderlings, laying dates could not (with a few exceptions) be determined for tracked individuals, as geolocators stopped functioning prior to breeding or birds did not breed. To analyse trends in laying dates for these populations, we supplemented our dataset with laying dates determined from field data for the same study sites and years (Table 1).

### 2.5 Date of Snowmelt

Snow cover for all study sites was estimated using satellite images of the 500 m resolution MODIS Terra Surface Reflectance Daily Global product [MOD09GA, v6.1, (Vermote and Wolfe 2021)]. The analysis was conducted in Google Earth Engine (Gorelick et al. 2017), using the R‐package RGEE (Aybar et al. 2020) and the automated workflows for the quantification of snowmelt by Versluijs (2024).

We manually mapped spatial overlays of study sites (Table S1). Although sites differed strongly in size, all sites represented areas of mostly homogeneous elevation with little expected variation in snowmelt, thus giving an average across each of the study sites. We extracted satellite images between March 15 and September 15 for each study site covering years 2000–2023. Within each extracted image, we masked pixels that were classified as clouds by the 1000 m MOD35 cloud mask product (MODIS Atmosphere Science Team 2012) and as waterbodies (i.e., oceans, rivers, lakes and large ponds) by the 250 m Terra Land Water Mask dataset (MOD44W, v6.0, Carroll et al. 2017). We subsequently calculated the normalised difference snow index (i.e., NDSI; Dietz et al. 2012; Dozier 1989) value per pixel, as the normalised difference between the green and the short‐wave infrared band:

$$\text{NDSI}=\frac{\left({\text{Green}}_{B04}-{\text{SWIR}}_{B06}\right)}{\left({\text{Green}}_{B04}+{\text{SWIR}}_{B06}\right)}$$

For each satellite image and within each study site, we then calculated the percentage of snow cover as the fraction of pixels with NDSI values larger than 0.4 (Dozier 1989; Hall et al. 1995). We fit a general additive model (GAM; Wood 2023) to these annual time series of snow cover and extracted the moment the GAM first dropped below 50% snow cover, which we used in subsequent analyses as the date of snowmelt.

### 2.6 Statistical Analyses

For analyses, we used generalised linear mixed models [GLMMs, using r‐package ‘lme4’ (Bates et al. 2012)] including a random intercept for individual birds in models with year as the independent variable and random intercepts for individual birds and year in all other models. The year and date of the snowmelt were centred by subtracting population‐specific means. As strong interannual variation in environmental conditions (incl. date of snowmelt) could distort temporal trends, we excluded short time series (less than 5 years) from analyses of trends over time (i.e., over the years). Below, the steps of the full analysis are described in detail:

First, we analysed trends in the date of snowmelt over time (using the specific time period for which tracking data for the population was available) using linear models (LMs) including the date of snowmelt as the dependent variable and year, population and their interaction as independent variables (i).

- date of snowmelt (*D*_sm_) = intercept (intrcpt) + year (*Y*) + population (*P*) + *P* × *Y*

Thereafter, we analysed the relationships of arrival date and laying date with the date of snowmelt (ii, iv) and trends over time (iii, v). We used GLMMs for all species combined, including arrival date or laying date as a dependent variable and either date of snowmelt, population and their interaction, or year, population and their interaction as independent variables. From these GLMMs, population‐specific trends were inspected post hoc using confidence intervals in the ‘emtrends’ function in the package ‘emmeans’ (Lenth 2017).

ii arrival date (Da) = *D*_sm_ + *P* + *D*_sm_ × *P* + (1|ID) + (1|*Y*)
iii Da = *Y* + *P* + *Y* × *P* + (1|ID)
iv laying date (Dl) = *D*_sm_ + *P* + *D*_sm_ × *P* + (1|ID) + (1|*Y*)
v Dl = *Y* + *P* + *Y* × *P* + (1|ID)

Subsequently, we analysed whether population‐level relationships between arrival date and snowmelt, as well as population‐level trends over time, could be explained by similar changes in departure date, migration distance and travel speed in relation to snowmelt or with time. To do so, we extracted slopes from population‐specific GLMMs (vi) with arrival date, departure date, migration distance, or travel speed as dependent variables and date of snowmelt as an independent variable, including random intercepts as described above. In these GLMMs, arrival date, departure date, migration distance and travel speed were standardised by subtracting the overall mean and dividing by the standard deviation to allow for a later comparison of slopes. We then fitted LMs (vii) for all populations combined with arrival date slope as the dependent variable and departure date, migration distance and travel speed slopes as independent variables. We followed the same procedure for population‐level trends with time, extracting slopes from population‐specific GLMMs with time as an independent variable (viii) and fitting LMs for all populations combined (ix).

vi Da/departure date (Dd)/migration distance (MD)/travel speed (*V*) = *D*_sm_ + (1|ID) + (1|*Y*)
vii slope arrival date ~ date of snowmelt (Da_sm_) = intrcpt + slope departure date (Dd_sm_) + slope migration distance (MD_sm_) + slope travel speed (*V*_sm_)
viii Da/Dd/MD/*V* = *Y* + (1|ID)
ix slope arrival date–year (Day) = intrcpt + Dd_y_ + MD_y_ + V_y_.

Finally, we analysed whether variation in laying dates between individuals and among years could be explained either by local date of snowmelt or individual arrival date, using GLMMs (x) including date of egg laying as the dependent variable, and date of snowmelt, arrival date and population, as well as interactions date of snowmelt × population and arrival date × population, as independent variables, along with random intercepts described above. In these analyses, laying and arrival dates were centred by subtracting population‐specific means.

(x) Dl = Dsm + Da + P + Dsm × *P* + Da × *P* + (1|*Y*) + (1|*P*)

We used AICc values to compare the performance of models including all possible combinations of independent variables, including an intercept‐only model. For most analyses, this meant comparing the performance of models including either the date of snowmelt or the year as independent variables with intercept‐only models. We selected the model with the lowest AICc, but models within 2 ΔAICc of the top supported model were also considered informative, except when containing additional parameters (Arnold 2010). All analyses were conducted using R (version 4.3.1).

### 2.7 Data From Literature

We collated papers which reported on arrival and laying date of arctic migratory birds relative to the date of snowmelt in the Web of Science database, conducted on 7 February 2024. We used the search term: bird AND Arctic AND (snowmelt OR snow cover OR snow) AND (migration timing OR arrival OR laying date OR nest initiation OR egg) using the option ‘all fields’. This resulted in 213 papers, which were scanned for statistics and data on the timing of migratory arrival (on northern stopover sites or breeding sites), measured for at least 3 years in relation to any metric of snow cover. We omitted studies where it was not possible to extract slopes, arrival, laying, or snowmelt dates from either the text, tables, or graphs. Data were found in six papers (Table S2). Three papers (which were not identified in the search) known to contain similar data were added (Ely et al. 2018; Hupp et al. 2018; Lameris et al. 2018). Unpublished data for three additional species were supplemented by S. Volkov, with the data provided in the dataset linked to this paper (Lameris et al. 2025) and data collection following methods described in Volkov and Pozdnyakov (2021). Among all considered studies, the snow cover fraction used or extracted as a measure of ‘date of snowmelt’ varied between 60% and 0%. In general different fractions of snow cover will be highly correlated (e.g., 0.99 Pearson's correlation between 0.25 and 0.75 fraction snow cover in our own dataset), although the moment of 0% snow cover may show less of a correlation with mid‐point snow cover. In total, the dataset from the literature contained 18 species (five species of waterfowl, 10 shorebirds, two passerines and one gull) from six different locations in the Holarctic (Table S2). We extracted slopes of the change in arrival dates, laying dates and date of snowmelt over time, and slopes of the change in arrival dates and laying dates in relation to date of snowmelt. We then used *t*‐tests to analyse whether the slope in the change in arrival date and laying date, with time as well as in relation to the date of snowmelt, differed from zero. In addition, we used LMs to analyse whether changes in arrival and laying date over time correlated with changes in the date of snowmelt over time, comparing models with either slope of arrival or laying date as the dependent variable and the slope of date of snowmelt as an independent variable, and performing model selection as described above.

## 3 Results

### 3.1 Trends in Date of Snowmelt

The date of snowmelt generally advanced over the years, although the null model was competitive (−0.82 ± 0.43 mean slope coefficient ± standard deviation, units days per year, models without year = 1.4 ΔAICc compared to model with year). This trend did not differ between populations (models with interaction term population x year = 44.1 ΔAICc compared to the model without).

### 3.2 Population‐Level Trends in Timing of Arrival and Laying

**FIGURE 2:**
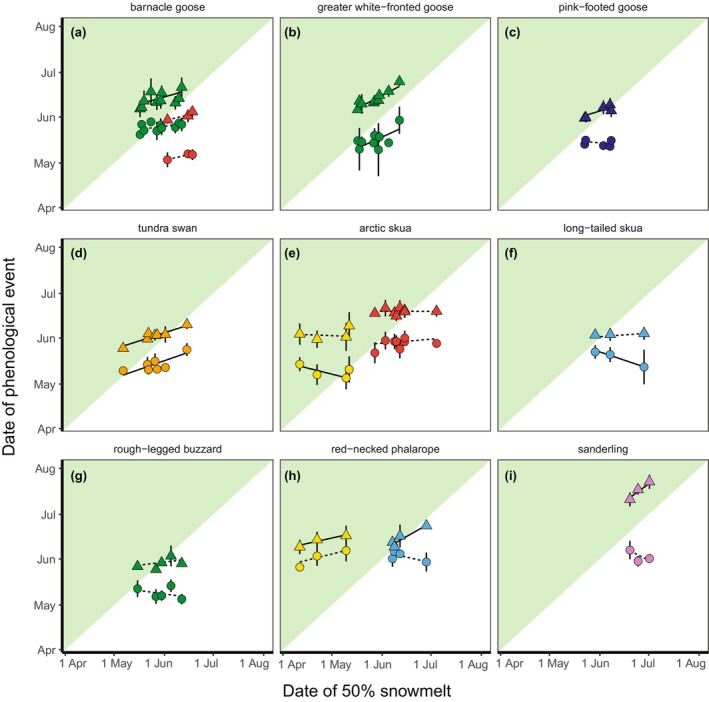
Annual average date of arrival in the Arctic (dots) and date of egg‐laying (triangles) in relation to the date of 50% snowmelt at the breeding site per species and breeding site (a‐i), with vertical lines showing standard deviations. The white–green transition shows the *y* = *x* line where date of arrival or egg‐laying equals date of 50% snowmelt. Symbols are coloured according to study sites, as in Figure 1. Lines show slopes (output from GLMMs) in the date of arrival or egg‐laying with day of 50% snowmelt, solid lines for slopes where confidence intervals did not overlap with 0, dashed lines for slopes overlapping with 0.

Arrival date in the Arctic was related to the date of snowmelt on the breeding grounds (model with population only = 26.8 ΔAICc compared to the model with date of snowmelt, population and interaction, Table S3) and differed between populations (model without interaction = 30.5 ΔAICc compared to a model with, Table S3). The confidence intervals of most population‐specific trends overlapped with zero (Table 2; Figure 2), with the exception of four populations. Greater white‐fronted geese and tundra swans arrived earlier in years with early snowmelt at their breeding grounds (greater white‐fronted geese: 0.50 ± 0.11, 95% CI [0.28–0.72], tundra swans: 0.38 ± 0.08, 95% CI [0.22–0.54]). Arctic skuas breeding in Slettnes and long‐tailed skuas arrived later in years with earlier snowmelt (Arctic skua: −0.27 ± 0.08, 95% CI [−0.43 to −0.12]; long‐tailed skua: −0.36 ± 0.08, 95% CI [−0.53 to −0.21]).

**TABLE 2:** Slopes and standard deviations of trends in arrival date and laying date over date of snowmelt as well as over time at the population level.

Population	Arrival–snowmelt	Laying date–snowmelt	Snowmelt–time	Arrival–time	Laying date–time
*Barnacle goose (Kolguev Island)*	0.14 ± 0.10 [−0.05, 0.33]	**0.31 ± 0.10 [0.12, 0.50]**	**−1.01 ± 0.54 [−2.11, 0.09]**	**−0.32 ± 0.18 [−0.68, 0.04]**	*−* **0.41 ± 0.19 [−0.78, −0.04]**
*Barnacle goose (Kongsfjorden)*	0.25 ± 0.21 [−0.17, 0.67]	0.26 ± 0.18 [−0.10, 0.61]	**−1.37 ± 6.47 [−14.45, 11.71]**		
*Greater white‐fronted goose*	**0.50 ± 0.11 [0.27, 0.73]**	**0.57 ± 0.10 [0.37, 0.77]**	**−1.89 ± 1.11 [−4.12, 0.34]**	**−0.73 ± 0.40 [−1.54, 0.01]**	**−1.71 ± 0.36 [−2.42, −1.00]**
*Pink‐footed goose*	−0.10 ± 0.15 [−0.40, 0.21]	**0.34 ± 0.13 [0.07, 0.60]**	**−1.02 ± 2.89 [−6.87, 4.83]**	**−1.04 ± 0.83 [−2.64, 0.55]**	0.59 ± 0.70 [−0.80, 1.98]
*Tundra swan*	**0.38 ± 0.08 [0.22, 0.54]**	**0.35 ± 0.13 [0.10, 0.61]**	**−3.61 ± 1.73 [−7.11, −0.12]**	**−1.01 ± 0.39 [−1.79, −0.23]**	**−**1.84 ± 1.08 [−3.96, 0.29]
*Red‐necked phalarope (Ammarnäs)*	−0.25 ± 0.19 [−0.63, 0.12]	**0.62 ± 0.11 [0.40, 0.84]**	**−0.58 ± 4.09 [−8.85, 7.70]**		
*Red‐necked phalarope (Slettnes)*	0.27 ± 0.18 [−0.09, 0.63]	**0.24 ± 0.07 [0.09, 0.39]**	**14.26 ± 6.47 [1.18, 27.34]**		
*Sanderling*	−0.54 ± 0.39 [−1.30, 0.22]	**0.80 ± 0.14 [0.53, 1.07]**	**−2.85 ± 4.24 [−11.41, 5.71]**		
*Arctic skua (Kongsfjorden)*	0.09 ± 0.10 [−0.10, 0.28]	0.00 ± 0.09 [−0.18, 0.17]	**0.40 ± 1.41 [−2.46, 3.25]**	**−0.52 ± 0.30 [−1.12, 0.07]**	−0.47 ± 0.40 [−1.27, 0.32]
*Arctic skua (Slettnes)*	**−0.27 ± 0.08 [−0.43, −0.12]**	−0.04 ± 0.08 [−0.20, 0.11]	**10.91 ± 4.09 [2.64, 19.18]**		
*Long‐tailed skua*	**−0.37 ± 0.08 [−0.53, −0.21]**	0.08 ± 0.09 [−0.11, 0.27]	**15.03 ± 6.47 [1.95, 28.11]**		
*Rough‐legged buzzard*	−0.15 ± 0.18 [−0.50, 0.20]	0.13 ± 0.18 [−0.22, 0.48]	**1.57 ± 2.89 [−4.28, 7.42]**	***−*1.72 ± 1.13 [−3.98, 0.53]**	0.33 ± 1.30 [−2.24, 2.98]

*Note:* Bird trends over time are only analysed for populations for which more than 5 consecutive years are available. Trends marked in bold indicate when 95% confidence intervals did not overlap with 0. Trends in snowmelt date and arrival date–time are all marked in bold as the best model showed a common trend for all populations.

**FIGURE 3:**
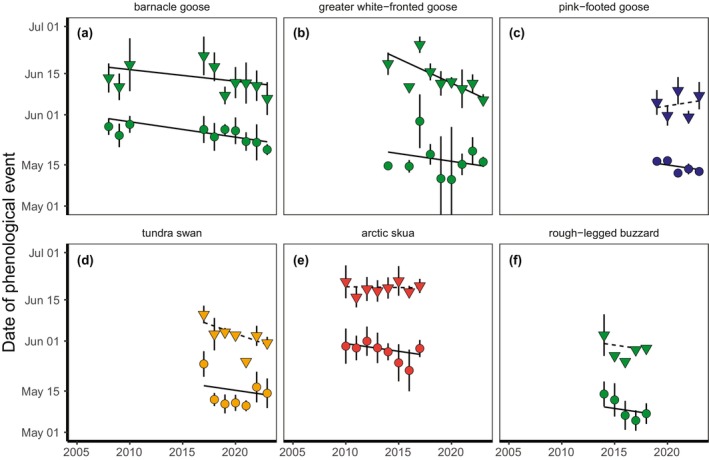
Annual average date of arrival in the Arctic (dots) and date of egg‐laying (triangles) with time, with vertical lines showing standard deviations for six Arctic populations (a‐f) with data for at least 5 years. Symbol and line colours and shapes are as described in Figure 2. Lines show slopes (output from GLMMs) in the date of arrival/egg‐laying with time.

Arrival date in the Arctic advanced with time (0.52 ± 0.13 days earlier arrival per year, a model with a population only = 11.0 ΔAICc compared to a model with year and population, Figure 3) which did not vary between populations (model with interaction = 2.7 ΔAICc compared to model without, Table S3).

Egg‐laying dates advanced with earlier snowmelt (model with a population only = 83.0 ΔAICc compared to model with population, date of snowmelt and their interaction; Figure 2, Table S3). Again, the effect of snowmelt differed between populations (model without interaction = 25.4 ΔAICc compared to model with). Confidence intervals did not overlap with 0 for several populations, showing earlier laying dates with earlier snowmelt for red‐necked phalaropes (Ammarnäs: 0.62 ± 0.11, 95% CI [0.40–0.84]; Slettnes: 0.24 ± 0.08, 95% CI [0.09–0.39]), sanderlings (0.80 ± 0.14, 95% CI [0.53–1.07]), barnacle geese (Kolguev Island, 0.31 ± 0.10, 95% CI [0.12–0.50]), greater white‐fronted geese (0.57 ± 0.10, 95% CI [0.38–0.77]), pink‐footed geese (0.34 ± 0.13, 95% CI [0.07–0.60]) and tundra swans (0.35 ± 0.13, 95% CI [0.10–0.60], Table 2).

Egg‐laying dates advanced with time (model with a population only = 20.5 ΔAICc compared to a model with population, year and their interaction, Figure 3), and this response also differed between populations (model without interaction = 9.4 ΔAICc compared to model with, Table S3). Confidence intervals of most populations overlapped with 0, with the exception of barnacle geese (Kolguev Island, −0.41 ± 0.19, 95% CI [−0.78 to −0.04]) and greater white‐fronted geese (−1.71 ± 0.36, 95% CI [−2.42 to −1.00]).

### 3.3 Factors Explaining Population‐Level Trends in Arrival Timing

**FIGURE 4:**
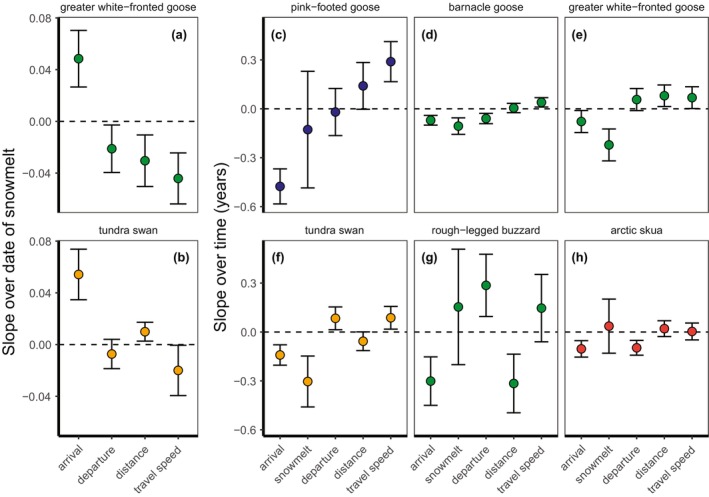
Slopes (±standard deviations) in arrival date compared to the slopes in non‐breeding site departure date, travel distance, and travel speed (the three potential explanatory mechanisms), over date of snowmelt (a,b) and trends with time (c‐h). Trends in date of snowmelt with time are also shown in (c‐h). The raw values (i.e., arrival date, snowmelt date, departure date, travel distance and travel speed) were centred and standardised to make the slope values suitable for quantitative comparison. Colours show different breeding sites, as in Figure 1. The horizontal dotted line shows the 0 intercept.

Slopes of arrival date over the date of snowmelt were not explained by slopes of departure, distance, or travel speed over snowmelt (model with travel speed = 0.6 ΔAICc compared to the intercept‐only model, Table S4). For greater white‐fronted geese and tundra swans, which both advanced arrival in years with earlier snowmelt (see above), earlier arrival appeared to be associated with later departure, longer distance and higher travel speeds (greater white‐fronted goose, Figure 4a) and shorter migration distance and higher travel speeds (tundra swan, Figure 4b), respectively. Populations that showed earlier arrival date with time also showed higher travel speed with time (intercept‐only model = 4.8 ΔAICc compared to model with travel speed, Table S4, Figure 4c–h), specifically in pink‐footed geese, barnacle geese, white‐fronted geese and tundra swans.

### 3.4 Timing of Egg‐Laying in Relation to Arrival

Variation in individual egg‐laying dates was explained both by date of snowmelt (0.22 ± 0.05 days earlier egg‐laying per earlier day of snowmelt, model with arrival date and population = 15.1 ΔAICc compared to model with date of snowmelt, arrival date and population, Table S5) as well as arrival date (0.20 ± 0.05 days earlier egg‐laying per earlier day of arrival, a model with the date of snowmelt and population = 8.6 ΔAICc compared to a model with arrival date, date of snowmelt and population). The date of snowmelt explained more variation compared to the arrival date (model with arrival date and population = 7.7 ΔAICc lower compared to a model with the date of snowmelt and population).

### 3.5 Data From Literature

**FIGURE 5:**
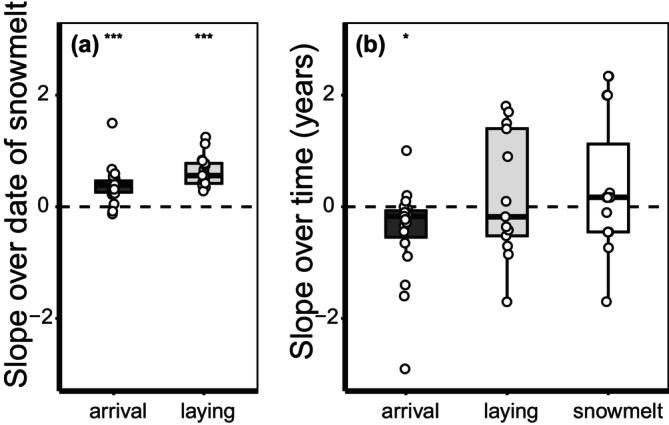
Boxplots summarising the data extracted from literature, showing the variation in slopes of arrival date (dark grey), laying date (light grey) in relation to date of snowmelt (a) and slopes in arrival date, laying date and date of snowmelt (white) as trends with time (b), with stars denoting significance levels (*p < 0.05, ***p < 0.0005).

In studies retrieved from the literature, arrival and laying dates advanced with the date of snowmelt, although laying dates advanced on average faster (0.62 days advance per earlier day of snowmelt, *t* = 9.39, *p* < 0.001) than the arrival date (0.39 days advance, *t* = 6.03, *p* < 0.001, Figure 5). Arrival dates also advanced over time (0.47 days advance per year, *t* = −2.25, *p* = 0.037, Figure 5), but laying dates did not (*t* = 0.66, *p* = 0.52, Figure 5). On average, the date of snowmelt did not advance in these studies (*t* = 1.50, *p* = 0.15, Table S2) and earlier laying dates, but not arrival dates with time were explained by trends in the date of snowmelt (laying dates: intercept‐only model = 15.5 ΔAICc compared to model with date of snowmelt; arrival dates: model with date of snowmelt = 1.4 ΔAICc compared to intercept‐only model).

## 4 Discussion

Trends in the date of snowmelt were highly variable between populations (standard deviation of 0.39 for a slope of −0.83), yet in general, snowmelt appeared to advance with time. Arctic migratory bird populations showed varying responses in arrival timing with earlier snowmelt, and only two out of 12 populations, both waterfowl species (greater white‐fronted geese and tundra swans), advanced their arrival with an earlier date of snowmelt. At the same time, most populations did advance egg‐laying dates in years with earlier snowmelt (seven out of 12 populations). With time, populations advanced the timing of arrival, but not in all cases the date of egg‐laying. To explain these patterns, we discuss responses in arrival and laying dates in the context of environmental and internal constraints.

### 4.1 Earlier Arrival in the Arctic

In years with early snowmelt, an advancement in laying dates is facilitated by a similar advancement in the timing of arrival (as we find that laying dates are partially explained by arrival timing) which shows that earlier arrival is an important prerequisite for earlier laying dates. As such, we would expect advancements in arrival in years with earlier snowmelt, which would aid individuals in advancing their laying dates. We found only two out of 12 populations, both waterfowl species, to advance arrival with earlier snowmelt. This suggests that for most populations, a flexible response in arrival timing to annually varying environmental conditions is either not possible or not beneficial, as birds face environmental or internal limitations. Environmental barriers, such as the large stretch of ocean between the European mainland and Svalbard (Geisler et al. 2022), may limit terrestrial birds to predict environmental conditions on their breeding grounds (Kölzsch et al. 2015). For comparison, the two waterfowl populations that did show adjustment of arrival timing to date of snowmelt (greater white‐fronted geese and tundra swans) used relatively many stopovers along the way [on average per individual, 3.0 ± 1.6 for greater white‐fronted geese and 3.4 ± 1.3 for tundra swans; see also (Nuijten and Nolet 2020; van Wijk et al. 2012)], which is more stopovers than other populations in our dataset (Table S3). The use of multiple stopovers will allow birds to gradually assess the progression of spring onset while at the same time offering more options to depart earlier.

Earlier migrations may be further limited by internal constraints, as earlier departures from stopover sites will come at the cost of (re)fuelling. When birds travel with only enough energy stores to fuel a flight to the next stopover site, shorter stays will only be possible if birds can also fuel faster (Lindström et al. 2019), as they otherwise do not have the stores to reach their next destination. While the limited number of populations adjusting arrival to annually varying snowmelt could suggest that internal constraints play an important role in limiting earlier arrival, our result on advancements in arrival with time in populations with long time series shows otherwise. Apparently, many of our study populations are able to make advancements in arrival in the Arctic with time, which is further reinforced by similar trends in the data that we extracted from the literature. Earlier arrival with time is mainly explained by faster migrations, further showing that birds are not lacking in body stores to spend less time on stopover sites. Many of the populations in our long‐term dataset are large waterfowl species that are, at least to some extent, capital breeders (Hahn et al. 2011; Klaassen et al. 2017; Nolet 2006), carrying body stores to not only sustain migratory flight but partially also reproduction (Kölzsch et al. 2016). Using these stores to sustain extended migratory flight instead, would allow them to increase travel speed by spending less time on stopover sites. At the same time, this will reduce body stores with which they arrive on the breeding grounds and which they can use for reproduction, as previously shown for barnacle geese (Lameris et al. 2018). Yet, our results show that even Arctic skuas and rough‐legged buzzards, which are capital breeders to a lesser extent (Hobson et al. 2000), appear able to advance arrival with time, suggesting that other factors such as improved feeding conditions during migration may play an additional role. When faster migrations go hand in hand with earlier departure from stopover sites and therefore shorter fuelling times, it is important to consider that this may not be without consequences, as it can lead to reduced survival (Rakhimberdiev et al. 2018) or delays in breeding after arrival (Lameris et al. 2018).

### 4.2 Earlier Laying Dates

A remaining question is whether populations that do not adjust migration timing to local environmental conditions are, as a result, limited in advancing their laying dates. We find that all populations in our dataset, except one waterfowl and shorebird population, advanced laying dates with earlier snowmelt but not with time. Similarly, our analysis of data from the literature showed a similar pattern of laying dates strongly advancing with earlier snowmelt, and with time only when date of snowmelt also advanced. A response of laying dates to year‐to‐year variation in environmental conditions is found for many other similar populations, both in the Arctic (Lameris et al. 2019; Liebezeit et al. 2014; Schmidt et al. 2023; Smith et al. 2010; Tavera et al. 2024) as well as in many other seasonal environments (Crick et al. 1997; Visser et al. 2012; Zhemchuzhnikov et al. 2021). Populations that adjust laying dates to the date of snowmelt but not their arrival date show an increasing interval between arrival and laying in years with late snowmelt. This suggests that birds delay breeding when the snow melts late, waiting until breeding patches become snow‐free or food becomes available (Meltofte et al. 2007; Prop and de Vries 1993; Smith et al. 2010); yet our results show that the arrival date may pose an additional limitation to earlier breeding. Furthermore, multiple populations in our study show similar advancements in arrival and laying dates, suggesting a strong link in timing between these processes. One would need to experimentally advance arrival timing in order to fully confirm whether timing in arrival constrains the laying date.

At the same time, the lack of a response in laying date to annually varying conditions in both skuas and in rough‐legged buzzards may not be explained by a similar lack in response of arrival dates. Strong phenological adjustments are likely to be more profitable for organisms at lower trophic levels, feeding on primary producers and primary consumers that typically advance quickly in response to climate warming (Thackeray et al. 2010, 2016). In contrast, organisms at higher trophic levels may have less benefit of breeding early due to an already slower phenological response of their prey, which may explain a limited response. Rough‐legged buzzards on Kolguev Island prey mostly on waterfowl offspring (Curk et al. 2022) and may not need to advance laying dates to have access to prey throughout their breeding season, even if waterfowl advance laying dates. Long‐tailed skuas, which also did not adjust laying dates to the date of snowmelt, depend on their reproductive success and nesting on available rodent prey, which can vary significantly from year to year (Barraquand et al. 2014; Gilg et al. 2006), but may be less related to the timing of snowmelt or other temperature‐driven processes (Grabowski et al. 2013). Finally, the studied population of Arctic skuas is mainly kleptoparasites of other seabirds, which generally do not advance their timing of breeding over time or in response to temperature (Keogan et al. 2018).

### 4.3 Long‐Term Responses in Arrival

The analysis of tracking data as well as data from literature shows trends towards earlier arrival dates in the Arctic, even if this does not always coincide with earlier snowmelt. Furthermore, long‐term advancements in laying dates do not appear to be a general pattern in our dataset or the data extracted from the literature. This suggests that birds respond to other factors or general trends in environmental conditions and could even imply a response to long‐term climatic conditions. Birds may show a trend towards earlier arrival as they experience a lower risk of arriving too early, before snowmelt and while food is still not available (Prop and de Vries 1993). In addition, changes in environmental conditions along the migratory flyways might also enable earlier migrations (Ouwehand et al. 2023). Non‐breeding sites as well as stopover sites may have become more benign for migrants in terms of climate and, especially for herbivorous waterfowl, food availability. This could facilitate earlier departures and spending the non‐breeding period closer to the breeding grounds, but also more rapid energy deposition prior to migratory departure (Lindström et al. 2019), which would allow the skipping of subsequent stopover sites (Eichhorn et al. 2009). Such changes in the migratory strategies of individual birds may rapidly spread through the population if individuals learn from each other (Oudman et al. 2020; Tombre et al. 2019). Earlier arrival will benefit reproduction in general, even if birds do not advance laying dates, due to better chances for territory or mate acquisition (Drent et al. 2003; Kokko 1999), as well as gathering energy stores which could benefit reproduction (Boom, Schreven, et al., 2023; Hupp et al. 2018).

### 4.4 Additional Factors

Several additional factors might partially explain the trends and variations in our study. First of all, the variation in size of study sites and potentially resulting variation in snowmelt patterns could also affect the relationships found. Specifically for the smallest study sites, for example the breeding islands of barnacle geese and Arctic skuas in Kongsfjorden on Svalbard, a limited number of pixels are available to analyse snow cover (Table S1). This could lead to inaccuracies in the date of snowmelt and, as such, affect the likelihood of finding relationships with migration and reproduction timing.

We find that, although the arrival date in the Arctic correlated with the date of snowmelt, the arrival date significantly advanced only for two populations of waterfowl. Potentially, the location at which populations cross the Arctic Circle—our measure/proxy for arrival date—may still be too far from the eventual breeding locations to predict conditions at breeding sites. When we compare the timing of arrival at the actual breeding site in relation to the date of snowmelt, which was possible for six populations with GPS tracks (Figure S2; Table S3; Supporting Information Methods), we find only one additional population, barnacle geese breeding on Kolguev Island, that arrived earlier in years with early snowmelt. This is in accordance with earlier findings for this population (Lameris et al. 2018). Three other populations, the barnacle geese breeding in Kongsfjorden, rough‐legged buzzards and pink‐footed geese, do not show adjustments in arrival date at their respective breeding grounds with earlier snowmelt (Figure S1). In addition, long‐tailed skuas arrived later in years when snow melted earlier. This counterintuitive result could perhaps be explained by a strong delay in the date of snowmelt over the 3 years over which this population was tracked (Figure S1), with birds thus also showing strong advancement of arrival date with time.

Concerning trends of arrival over time, we have so far not considered individual effects. Individual migrations may become more efficient with increasing age or selective disappearance of individuals with later migration phenology (Aikens et al. 2024; Sergio et al. 2014), as well as learned experience about optimal migration timing, that is, earlier arrival, to achieve high reproductive success. For most populations, with the exception of pink‐footed geese and long‐tailed skuas, new individuals were tagged in almost every year of the study. Therefore, while individual changes could explain results to some extent, such changes will also have been present at the population level.

## 5 Conclusions

Our study shows that migratory birds advance their arrival in the Arctic. Earlier arrival in response to annually varying environmental conditions may only be possible for populations, such as some waterfowl populations, which make several stopovers on the way from which they can predict breeding site conditions. At the same time, a range of migratory species (incl. four waterfowl populations, Arctic skua and rough‐legged buzzard) is able to advance arrival, explained mostly by faster migrations and thus less time spent on stopovers. Advancements in arrival timing may be facilitating simultaneous trends in earlier laying dates, both in years with earlier snowmelt and with time. These trends with time perhaps are responses to a general trend in earlier springs. An important consideration is that faster migrations by earlier departure from stopover sites may not be without consequences and can result in reduced survival (Rakhimberdiev et al. 2018) or delays in breeding after arrival (Lameris et al. 2018). Nevertheless, our results suggest considerable flexibility in migration timing behaviour which allows birds to rapidly respond to climate warming.

## Author Contributions

Thomas K. Lameris and Rob S.A. van Bemmelen conceived the idea for this study; Thomas K. Lameris, Michiel P. Boom, Rascha J.M. Nuijten, Götz Eichhorn, Bruno J. Ens, Klaus‐Michael Exo, Petr M. Glazov, Sveinn Are Hanssen, Philip Hunke, Henk P. van der Jeugd, Margje E. de Jong, Andrea Kölzsch, Alexander Kondratyev, Helmut Kruckenberg, Olga Kulikova, Hans Linssen, Julia A. Loshchagina, Maarten J.J.E. Loonen, Jesper Madsen, Børge Moe, Sander Moonen, Gerhard J.D.M. Müskens, Bart A. Nolet, Ivan Pokrovsky, Jeroen Reneerkens, Isabella B.R. Scheiber, Hans Schekkerman, Kees H.T. Schreven, Ingrid Tulp, Mo A. Verhoeven, Tom S.L. Versluijs, Sergey Volkov, Martin Wikelski and Rob S.A. van Bemmelen contributed to data collection; Helmut Kruckenberg, Bart A. Nolet, Jeroen Reneerkens, Isabella B.R. Scheiber and Martin Wikelski obtained funding; Thomas K. Lameris analysed the data; Thomas K. Lameris wrote the manuscript; and all other authors contributed to the writing and approved the final manuscript.

## Ethics Statement

All research was conducted under permission from national committees on animal experimental work, following approved protocols by the Netherlands Institute of Ecology (NIOO13.14, NIOO 14.07), Centrale Commissie Dierproeven (permits 2016518, 20173788 and 202114712), Norwegian Food Safety Authority (FOTS id 2086, 23358, 29614, 3817, 5276, 6328, 6329, 7421, 8538, 15726) Jordbruksverket to Lund University, Sweden (permits M160‐11, M470‐12, M472‐12) and Lower Saxony State Office for Consumer Protection and Food Safety. Tagging of tundra swans in Lower Saxony, Germany, was carried out under the permission of ‘Niedersächsisches Landesamt für Verbraucherschutz und Lebensmittelsicherheit’ (LAVES; AZ 20/3603). Catching and tagging of Bewick's swans in the Netherlands was carried out under exemption of the Flora‐ en faunawet 75A, obtained through Dienst Regelingen (permit FF/75A/2016/044) and Wet natuurbescherming issued by Omgevingsdienst Brabant Noord (permits Z/046757 and Z/141920). No specific permissions were required for work on Rough‐legged Buzzards and Greater White‐fronted Geese on Kolguev Island from Federal Service for Supervision of Natural Resources (Rosprirodnadzor) according to §44 and §6 of the Federal Law of the Russian Federation No. 52 from 24.04.1995 (last update 08.08.2024) ‘On Wildlife’, as there were no Special Protected Natural Territories in our study area, and our activities did not include withdrawal of investigated species from nature. Capture and marking of pink‐footed geese in Svalbard was permitted by Mattilsynet (Norwegian animal research authority) to Aarhus University (reference no. 17/210528) and by the Governor of Svalbard (reference no. 17/01420‐4) and capture on Isdammen was permitted by Longyearbyen Lokalstyre (reference no. 2018/347‐5‐X70). Capture of barnacle geese was approved by the Governor of Svalbard (RIS ID 11237). For using GSM‐GPS transmitters on the territory of Russia, IP applied for and obtained permit No. 77–18/0854/4388 from The General Radio Frequency Centre, permit No. RU/2018/406 from the Federal Service for Supervision of Communications, Information Technology and Mass Media (Roskomnadzor), and permit No. RU0000045099 from the Federal Security Service; no permissions were required from the Federal Service for Technical and Export Control (FSTEC/FSTEK) according to Russian Federation government decree No. 633 from 29.08.2001 and Letter from FSTEK No. 240/33/1373 from 06.04.2015.

## Conflicts of Interest

The authors declare no conflicts of interest.

## Supporting information


Data S1.

## Data Availability

Tracking data used in this study are available from sources described and referenced in Table 1, including de Jong et al. 2024 (https://doi.org/10.1101/2024.08.30.610510), van der Jeugd et al. 2014 (https://doi.org/10.5441/001/1.ps244r11), Boom, Lameris, et al. 2023 (https://doi.org/10.1007/s00442‐023‐05386‐x), van Wijk et al. 2012 (https://doi.org/10.1111/j.1600‐0706.2011.20083.x), Kölzsch et al. 2016 (https://doi.org/10.1111/oik.03121), Schreven et al. 2021 (https://doi.org/10.1186/s40317‐021‐00249‐9), Nuijten and Nolet 2020 (https://doi.org/10.5751/ACE‐01682‐150214), Linssen et al. 2023 (https://doi.org/10.1111/gcb.16953), van Bemmelen et al. 2019 (https://doi.org/10.3389/fevo.2019.00086), Reneerkens et al. 2019 (https://doi.org/10.1111/1365‐2656.13118), van Bemmelen et al. 2024 (https://doi.org/10.1186/s40462‐024‐00459‐9), van Bemmelen et al. 2017 (https://doi.org/10.3354/meps12010) and Pokrovsky et al. 2021 (https://doi.org/10.1111/1365‐2656.13484). A complete dataset with dates of migratory arrival in the Arctic, egg‐laying dates and snowmelt for our study sites is available at Dryad Digital Repository in Lameris et al. 2025 (https://doi.org/10.5061/dryad.w0vt4b93d). Satellite images used to estimate snow cover were obtained from the NASA EOSDIS Land Processes Distributed Active Archive Center at https://doi.org/10.5067/MODIS/MOD09GA.061. Satellite images used to estimate cloudiness were obtained from the NASA EOSDIS Land Processes Distributed Active Archive Center at https://doi.org/10.5067/MODIS/MOD44W.006. Satellite images used to locate waterbodies were obtained from the NASA MODIS Adaptive Processing System, Goddard Space Flight Center at https://doi.org/10.5067/MODIS/MOD35_L2.006.

## References

[gcb70158-bib-0001] Aikens, E. O. , E. Nourani , W. Fiedler , M. Wikelski , and A. Flack . 2024. “Learning Shapes the Development of Migratory Behavior.” Proceedings of the National Academy of Sciences of the United States of America 121, no. 12: e2306389121. 10.1073/pnas.2306389121. 38437530 PMC10962998

[gcb70158-bib-0002] Alerstam, T. 2003. “Bird Migration Speed.” In Avian Migration, edited by P. Berthold , E. Gwinner , and E. Sonnenschein , 253–267. Springer Berlin Heidelberg. 10.1007/978-3-662-05957-9_17.

[gcb70158-bib-0003] Alerstam, T. , and J. Bäckman . 2018. “Ecology of Animal Migration.” Current Biology 28, no. 17: R968–R972. 10.1016/j.cub.2018.04.043. 30205072

[gcb70158-bib-0004] Alerstam, T. , M. Rosén , J. Bäckman , P. G. P. Ericson , and O. Hellgren . 2007. “Flight Speeds Among Bird Species: Allometric and Phylogenetic Effects.” PLoS Biology 5, no. 8: e197. 17645390 10.1371/journal.pbio.0050197 PMC1914071

[gcb70158-bib-0005] Arnold, T. W. 2010. “Uninformative Parameters and Model Selection Using Akaike's Information Criterion.” Journal of Wildlife Management 74, no. 6: 1175–1178. 10.2193/2009-367.

[gcb70158-bib-0006] Aybar, C. , Q. Wu , L. Bautista , R. Yali , and A. Barja . 2020. “rgee: An R Package for Interacting With Google Earth Engine.” Journal of Open Source Software 5, no. 51: 2272. 10.21105/joss.02272.

[gcb70158-bib-0007] Barraquand, F. , T. T. Høye , J. Henden , et al. 2014. “Demographic Responses of a Site‐Faithful and Territorial Predator to Its Fluctuating Prey: Long‐Tailed Skuas and Arctic Lemmings.” Journal of Animal Ecology 83, no. 2: 375–387. 10.1111/1365-2656.12140. 24128282

[gcb70158-bib-0008] Bates, D. , M. Maechler , and B. Bolker . 2012. “lme4: Linear Mixed‐Effects Models Using S4 Classes.” In *R package ver*., 0.999375‐32 [Computer software]. http://cran.r‐project.org/packagelme4.

[gcb70158-bib-0009] Bêty, J. , G. Gauthier , and J.‐F. Giroux . 2003. “Body Condition, Migration and Timing of Reproduction in Snow Geese: A Test of the Condition‐Dependent Model of Optimal Clutch Size.” American Naturalist 162, no. 1: 110–121. 10.1086/375680 12856240

[gcb70158-bib-0010] Boom, M. P. , H. P. V. D. Jeugd , B. Steffani , B. A. Nolet , K. Larsson , and G. Eichhorn . 2021. “Postnatal Growth Rate Varies With Latitude in Range‐Expanding Geese: The Role of Plasticity and Day Length.” Journal of Animal Ecology 91, no. 2: 417–427. 10.1111/1365-2656.13638. 34807466 PMC9300058

[gcb70158-bib-0011] Boom, M. P. , T. K. Lameris , K. H. T. Schreven , et al. 2023. “Year‐Round Activity Levels Reveal Diurnal Foraging Constraints in the Annual Cycle of Migratory and Non‐migratory Barnacle Geese.” Oecologia 202, no. 2: 287–298. 10.1007/s00442-023-05386-x. 37270441 PMC10307695

[gcb70158-bib-0012] Boom, M. P. , K. H. T. Schreven , N. H. Buitendijk , et al. 2023. “Earlier Springs Increase Goose Breeding Propensity and Nesting Success at Arctic But Not at Temperate Latitudes.” Journal of Animal Ecology 92, no. 12: 2399–2411. 10.1111/1365-2656.14020. 37899661

[gcb70158-bib-0013] Both, C. 2010. “Flexibility of Timing of Avian Migration to Climate Change Masked by Environmental Constraints En Route.” Current Biology 20, no. 3: 243–248. 10.1016/j.cub.2009.11.074. 20116248

[gcb70158-bib-0014] Both, C. , and M. E. Visser . 2001. “Adjustment to Climate Change Is Constrained by Arrival Date in a Long‐Distance Migrant Bird.” Nature 411, no. 6835: 296–298. 10.1038/35077063. 11357129

[gcb70158-bib-0015] Box, J. E. , W. T. Colgan , T. R. Christensen , et al. 2019. “Key Indicators of Arctic Climate Change: 1971–2017.” Environmental Research Letters 14, no. 4: 045010. 10.1088/1748-9326/aafc1b.

[gcb70158-bib-0016] Carroll, M. , C. DiMiceli , M. Wooten , A. Hubbard , R. Sohlberg , and J. Townshend . 2017. MOD44W MODIS/Terra Land Water Mask Derived From MODIS and SRTM L3 Global 250m SIN Grid V006 [Dataset]. NASA EOSDIS Land Processes Distributed Active Archive Center. 10.5067/MODIS/MOD44W.006.

[gcb70158-bib-0017] Chagnon‐Lafortune, A. , É. Duchesne , P. Legagneux , et al. 2024. “A Circumpolar Study Unveils a Positive Non‐linear Effect of Temperature on Arctic Arthropod Availability That May Reduce the Risk of Warming‐Induced Trophic Mismatch for Breeding Shorebirds.” Global Change Biology 30, no. 6: e17356. 10.1111/gcb.17356. 38853470

[gcb70158-bib-0018] Clausen, K. K. , J. Madsen , F. Cottaar , E. Kuijken , and C. Verscheure . 2018. “Highly Dynamic Wintering Strategies in Migratory Geese: Coping With Environmental Change.” Global Change Biology 24, no. 7: 3214–3225. 10.1111/gcb.14061. 29350875 PMC6032841

[gcb70158-bib-0019] Conklin, J. R. , P. F. Battley , M. A. Potter , and J. W. Fox . 2010. “Breeding Latitude Drives Individual Schedules in a Trans‐Hemispheric Migrant Bird.” Nature Communications 1, no. 1: 1–6. 10.1038/ncomms1072. 20842198

[gcb70158-bib-0020] Conklin, J. R. , S. Lisovski , and P. F. Battley . 2021. “Advancement in Long‐Distance Bird Migration Through Individual Plasticity in Departure.” Nature Communications 12, no. 1: 1–9. 10.1038/s41467-021-25022-7. PMC8346503 34362899

[gcb70158-bib-0021] Cooper, E. J. , S. Dullinger , and P. Semenchuk . 2011. “Late Snowmelt Delays Plant Development and Results in Lower Reproductive Success in the High Arctic.” Plant Science 180, no. 1: 157–167. 10.1016/j.plantsci.2010.09.005. 21421357

[gcb70158-bib-0022] Cramp, S. , and C. M. Perrins . 1988. Handbook of the Birds of Europe, the Middle East and Africa. The Birds of the Western Palearctic. Vol. 7. Oxford University Press.

[gcb70158-bib-0023] Crick, H. Q. P. , C. Dudley , D. E. Glue , and D. L. Thomson . 1997. “UK Birds Are Laying Eggs Earlier.” Nature 388, no. 6642: 526–527. 10.1038/41453.

[gcb70158-bib-0024] Curk, T. , O. Kulikova , I. Fufachev , M. Wikelski , K. Safi , and I. Pokrovsky . 2022. “Arctic Migratory Raptor Selects Nesting Area During the Previous Breeding Season.” Frontiers in Ecology and Evolution 10: 865482. 10.3389/fevo.2022.865482.

[gcb70158-bib-0025] de Jong, M. E. , A. J. Slettenhaar , R. W. Fokkema , et al. 2024. “Diel and Seasonal Rhythmicity in Activity and Corticosterone in an Arctic Migratory Herbivore: A Multifaceted Approach.” *bioRxiv*, 2024‐08. 10.1101/2024.08.30.610510.

[gcb70158-bib-0026] Dietz, A. J. , C. Kuenzer , U. Gessner , and S. Dech . 2012. “Remote Sensing of Snow—A Review of Available Methods.” International Journal of Remote Sensing 33, no. 13: 4094–4134. 10.1080/01431161.2011.640964.

[gcb70158-bib-0027] Dozier, J. 1989. “Spectral Signature of Alpine Snow Cover From the Landsat Thematic Mapper.” Remote Sensing of Environment 28: 9–22. 10.1016/0034-4257(89)90101-6.

[gcb70158-bib-0028] Drent, R. , C. Both , M. Green , J. Madsen , and T. Piersma . 2003. “Pay‐Offs and Penalties of Competing Migratory Schedules.” Oikos 103: 274–292.

[gcb70158-bib-0029] Egevang, C. , I. J. Stenhouse , R. a. Phillips , A. Petersen , J. W. Fox , and J. R. D. Silk . 2010. “Tracking of Arctic Terns *Sterna paradisaea* Reveals Longest Animal Migration.” PNAS 107, no. 5: 2078–2081. 10.1073/pnas.0909493107. 20080662 PMC2836663

[gcb70158-bib-0030] Eichhorn, G. , R. H. Drent , J. Stahl , A. Leito , and T. Alerstam . 2009. “Skipping the Baltic: The Emergence of a Dichotomy of Alternative Spring Migration Strategies in Russian Barnacle Geese.” Journal of Animal Ecology 78, no. 1: 63–72. 10.1111/j.1365-2656.2008.01485.x. 19120596

[gcb70158-bib-0031] Ely, C. R. , B. J. McCaffery , and R. E. Gill JR. 2018. “Shorebirds Adjust Spring Arrival Schedules With Variable Environmental Conditions: Four Decades of Assessment on the Yukon–Kuskokwim Delta, Alaska.” In Trends and Traditions: Avifaunal Change in Western North America, edited by W. D. Shuford , G. J. R. E. Gill , and C. M. Handel , 296–311. Western Field Ornithologists. 10.21199/SWB3.16.1.

[gcb70158-bib-0032] Evans, S. R. , and S. Bearhop . 2022. “Variation in Movement Strategies: Capital Versus Income Migration.” Journal of Animal Ecology 91, no. 10: 1961–1974. 35962601 10.1111/1365-2656.13800 PMC9825870

[gcb70158-bib-0033] Geisler, J. , J. Madsen , B. A. Nolet , and K. H. T. Schreven . 2022. “Sea Crossings of Migratory Pink‐Footed Geese: Seasonal Effects of Winds on Flying and Stopping Behaviour.” Journal of Avian Biology 2022, no. 10: e02985. 10.1111/jav.02985.

[gcb70158-bib-0034] Gilg, O. , B. Sittler , B. Sabard , et al. 2006. “Functional and Numerical Responses of Four Lemming Predators in High Arctic Greenland.” Oikos 113, no. 2: 193–216.

[gcb70158-bib-0035] Glazov, P. M. , J. A. Loshchagina , A. V. Kondratyev , E. M. Zaynagutdinova , H. Kruckenberg , and I. G. Pokrovsky . 2021. “The Long‐Term Monitoring of Bird Populations on Kolguev Island in the Barents Sea.” Arctic 74, no. 5: 23–40. 10.14430/arctic73845.

[gcb70158-bib-0036] Gorelick, N. , M. Hancher , M. Dixon , S. Ilyushchenko , D. Thau , and R. Moore . 2017. “Google Earth Engine: Planetary‐Scale Geospatial Analysis for Everyone.” Remote Sensing of Environment 202: 18–27. 10.1016/j.rse.2017.06.031.

[gcb70158-bib-0037] Grabowski, M. M. , F. I. Doyle , D. G. Reid , D. Mossop , and D. Talarico . 2013. “Do Arctic‐Nesting Birds Respond to Earlier Snowmelt? A Multi‐Species Study in North Yukon, Canada.” Polar Biology 36, no. 8: 1097–1105. 10.1007/s00300-013-1332-6.

[gcb70158-bib-0038] Hahn, S. , M. J. J. E. Loonen , and M. Klaassen . 2011. “The Reliance on Distant Resources for Egg Formation in High Arctic Breeding Barnacle Geese *Branta leucopsis* .” Journal of Avian Biology 42, no. 2: 159–168. 10.1111/j.1600-048X.2010.05189.x.

[gcb70158-bib-0039] Hall, D. K. , G. A. Riggs , and V. V. Salomonson . 1995. “Development of Methods for Mapping Global Snow Cover Using Moderate Resolution Imaging Spectroradiometer Data.” Remote Sensing of Environment 54, no. 2: 127–140. 10.1016/0034-4257(95)00137-P.

[gcb70158-bib-0040] Hobson, K. A. , J. Sirois , and M. L. Gloutney . 2000. “Tracing Nutrient Allocation to Reproduction With Stable Isotopes: A Preliminary Investigation Using Colonial Waterbirds of Great Slave Lake.” Auk 117, no. 3: 760–774. 10.1093/auk/117.3.760.

[gcb70158-bib-0041] Hupp, J. W. , D. H. Ward , D. X. Soto , and K. A. Hobson . 2018. “Spring Temperature, Migration Chronology, and Nutrient Allocation to Eggs in Three Species of Arctic‐Nesting Geese: Implications for Resilience to Climate Warming.” Global Change Biology 24, no. 11: 5056–5071. 10.1111/gcb.14418. 30092605

[gcb70158-bib-0042] Keogan, K. , F. Daunt , S. Wanless , et al. 2018. “Global Phenological Insensitivity to Shifting Ocean Temperatures Among Seabirds.” Nature Climate Change 8, no. 4: 313–317. 10.1038/s41558-018-0115-z.

[gcb70158-bib-0122] Kays, R. , S. C. Davidson , M. Berger , et al. 2022. “The Movebank System for Studying Global Animal Movement and Demography.” Methods in Ecology and Evolution 13, no. 2: 419–431.

[gcb70158-bib-0043] Kharouba, H. M. , and E. M. Wolkovich . 2020. “Disconnects Between Ecological Theory and Data in Phenological Mismatch Research.” Nature Climate Change 10, no. 5: 406–415. 10.1038/s41558-020-0752-x.

[gcb70158-bib-0044] Klaassen, M. , K. Abraham , R. Jefferies , and M. Vrtiska . 2006. “Factors Affecting the Site of Investment, and the Reliance on Savings for Arctic Breeders: The Capital–Income Dichotomy Revisited.” Ardea 94, no. 3: 371–384.

[gcb70158-bib-0045] Klaassen, M. , S. Hahn , H. Korthals , and J. Madsen . 2017. “Eggs Brought in From Afar: Svalbard‐Breeding Pink‐Footed Geese Can Fly Their Eggs Across the Barents Sea.” Journal of Avian Biology 48, no. 1: 173–179. 10.1111/jav.01364.

[gcb70158-bib-0046] Kokko, H. 1999. “Competition for early arrival in migratory birds.” Journal of Animal Ecology 68, no. 5: 940–950. 10.1046/j.1365-2656.1999.00343.x.

[gcb70158-bib-0047] Kölzsch, A. , S. Bauer , R. de Boer , et al. 2015. “Forecasting Spring From Afar? Timing of Migration and Predictability of Phenology Along Different Migration Routes of an Avian Herbivore.” Journal of Animal Ecology 84, no. 1: 272–283. 10.1111/1365-2656.12281. 25117616

[gcb70158-bib-0048] Kölzsch, A. , G. J. D. M. Müskens , H. Kruckenberg , et al. 2016. “Towards a New Understanding of Migration Timing: Slower Spring Than Autumn Migration in Geese Reflects Different Decision Rules for Stopover Use and Departure.” Oikos 125, no. 10: 1496–1507. 10.1111/oik.03121.

[gcb70158-bib-0049] Kondratyev, A. , E. Zaynagutdinova , and H. Kruckenberg . 2013. “Barnacle Goose *Branta leucopsis* Abundance on Kolguev Island—Current Status and History of Population Growth.” Wild 63, no. November: 56–71.

[gcb70158-bib-0050] Lameris, T. K. , M. P. Boom , R. J. M. Nuijten , et al. 2025. “Data From: Migratory Birds Advance Spring Arrival and Egg‐Laying in the Arctic, Mostly by Travelling Faster.” Dryad Digital Repository. 10.5061/dryad.w0vt4b93d.40201982

[gcb70158-bib-0051] Lameris, T. K. , M. E. de Jong , M. P. Boom , et al. 2019. “Climate Warming May Affect the Optimal Timing of Reproduction for Migratory Geese Differently in the Low and High Arctic.” Oecologia 191, no. 4: 1003–1014. 10.1007/s00442-019-04533-7. 31624958 PMC6853861

[gcb70158-bib-0052] Lameris, T. K. , F. Jochems , A. J. van der Graaf , M. Andersson , J. Limpens , and B. A. Nolet . 2017a. “Forage Plants of an Arctic‐Nesting Herbivore Show Larger Warming Response in Breeding Than Wintering Grounds, Potentially Disrupting Migration Phenology.” Ecology and Evolution 7, no. 8: 2652–2660. 10.1002/ece3.2859. 28428856 PMC5395431

[gcb70158-bib-0053] Lameris, T. K. , I. Scholten , S. Bauer , M. M. P. Cobben , B. J. Ens , and B. A. Nolet . 2017b. “Potential for an Arctic‐Breeding Migratory Bird to Adjust Spring Migration Phenology to Arctic Amplification.” Global Change Biology 23, no. 10: 4058–4067. 10.1111/gcb.13684. 28295932

[gcb70158-bib-0054] Lameris, T. K. , P. S. Tomkovich , J. A. Johnson , et al. 2022. “Mismatch‐Induced Growth Reductions in a Clade of Arctic‐Breeding Shorebirds Are Rarely Mitigated by Increasing Temperatures.” Global Change Biology 28: 829–847. 10.1111/gcb.16025. 34862835

[gcb70158-bib-0055] Lameris, T. K. , H. P. van der Jeugd , G. Eichhorn , et al. 2018. “Arctic Geese Tune Migration to a Warming Climate but Still Suffer From a Phenological Mismatch.” Current Biology 28: 2467–2473. 10.1016/j.cub.2018.05.077. 30033332

[gcb70158-bib-0056] Leingärtner, A. , J. Krauss , and I. Steffan‐Dewenter . 2014. “Elevation and Experimental Snowmelt Manipulation Affect Emergence Phenology and Abundance of Soil‐Hibernating Arthropods.” Ecological Entomology 39, no. 4: 412–418. 10.1111/een.12112.

[gcb70158-bib-0057] Liebezeit, J. R. , K. E. B. Gurney , M. Budde , S. Zack , and D. Ward . 2014. “Phenological Advancement in Arctic Bird Species: Relative Importance of Snow Melt and Ecological Factors.” Polar Biology 37, no. 9: 1309–1320. 10.1007/s00300-014-1522-x.

[gcb70158-bib-0058] Liebezeit, J. R. , P. A. Smith , R. B. Lanctot , et al. 2007. “Assessing the Development of Shorebird Eggs Using the Flotation Method: Species‐Specific and Generalized Regression Models.” Condor 109, no. 1: 32–47. 10.1650/0010-5422(2007)109[32:ATDOSE]2.0.CO;2.

[gcb70158-bib-0059] Lindström, Å. , T. Alerstam , and A. Hedenström . 2019. “Faster Fuelling Is the Key to Faster Migration.” Nature Climate Change 9, no. 4: 288–289. 10.1038/s41558-019-0443-7.

[gcb70158-bib-0060] Linssen, H. , E. E. Van Loon , J. Z. Shamoun‐Baranes , R. J. M. Nuijten , and B. A. Nolet . 2023. “Migratory Swans Individually Adjust Their Autumn Migration and Winter Range to a Warming Climate.” Global Change Biology 29, no. 24: gcb.16953. 10.1111/gcb.16953. 37795645

[gcb70158-bib-0061] Lisovski, S. 2018. “Light‐Level Geolocation in Polar Regions With 24‐Hour Daylight.” Wader Study 125, no. 2: 129–134. 10.18194/ws.00109.

[gcb70158-bib-0062] Lisovski, S. , C. M. Hewson , R. H. G. Klaassen , F. Korner‐Nievergelt , M. W. Kristensen , and S. Hahn . 2012. “Geolocation by Light: Accuracy and Precision Affected by Environmental Factors.” Methods in Ecology and Evolution 3, no. 3: 603–612. 10.1111/j.2041-210X.2012.00185.x.

[gcb70158-bib-0063] Lisovski, S. , B. J. Hoye , J. R. Conklin , et al. 2024. “Predicting Resilience of Migratory Birds to Environmental Change.” Proceedings of the National Academy of Sciences of the United States of America 121, no. 19: e2311146121. 10.1073/pnas.2311146121. 38648469 PMC11087779

[gcb70158-bib-0064] Lourenço, P. M. , R. Kentie , J. Schroeder , N. M. Groen , J. C. E. W. Hooijmeijer , and T. Piersma . 2011. “Repeatable Timing of Northward Departure, Arrival and Breeding in Black‐Tailed Godwits Limosa l. Limosa, but no Domino Effects.” Journal of Ornithology 152, no. 4: 1023–1032. 10.1007/s10336-011-0692-3.

[gcb70158-bib-0065] Maclean, I. M. D. , G. E. Austin , M. M. Rehfisch , et al. 2008. “Climate Change Causes Rapid Changes in the Distribution and Site Abundance of Birds in Winter.” Global Change Biology 14, no. 11: 2489–2500. 10.1111/j.1365-2486.2008.01666.x.

[gcb70158-bib-0066] Meltofte, H. , T. T. Høye , N. M. Schmidt , and M. C. Forchhammer . 2007. “Differences in Food Abundance Cause Inter‐Annual Variation in the Breeding Phenology of High Arctic Waders.” Polar Biology 30: 601–606. 10.1007/s00300-006-0219-1.

[gcb70158-bib-0067] Miller, M. R. , J. Y. Takekawa , J. P. Fleskes , et al. 2005. “Flight Speeds of Northern Pintails During Migration Determined Using Satellite Telemetry.” Wilson Bulletin 117, no. 4: 364–374.

[gcb70158-bib-0068] MODIS Atmosphere Science Team . 2012. MOD35_L2 MODIS/Terra Cloud Mask and Spectral Test Results 5‐Min L2 Swath 250m and 1km [Dataset]. NASA Level 1 and Atmosphere Archive and Distribution System Distributed Active Archive Center. 10.5067/MODIS/MOD35_L2.006.

[gcb70158-bib-0069] Morbey, Y. E. , and A. Hedenström . 2020. “Leave Earlier or Travel Faster? Optimal Mechanisms for Managing Arrival Time in Migratory Songbirds.” Frontiers in Ecology and Evolution 7: 492. 10.3389/fevo.2019.00492.

[gcb70158-bib-0070] Newton, I. 2009. “Moult and Plumage.” Ringing & Migration 24, no. 3: 220–226. 10.1080/03078698.2009.9674395.

[gcb70158-bib-0071] Nolet, B. A. 2006. “Speed of Spring Migration of Tundra Swans *Cygnus columbianus* in Accordance With Income or Capital Breeding Strategy?” Ardea 94, no. 3: 579–591.

[gcb70158-bib-0072] Nuijten, R. , and B. Nolet . 2020. “Chains as strong as the weakest link: remote assessment of aquatic resource use on spring migration by Bewick's Swans.” Avian Conservation and Ecology 15, no. 2: art14. 10.5751/ACE-01682-150214.

[gcb70158-bib-0073] Nuijten, R. J. M. , K. A. Wood , T. Haitjema , E. C. Rees , and B. A. Nolet . 2020. “Concurrent Shifts in Wintering Distribution and Phenology in Migratory Swans: Individual and Generational Effects.” Global Change Biology 26, no. 8: 4263–4275. 10.1111/gcb.15151. 32515077 PMC7384179

[gcb70158-bib-0074] Ockendon, N. , D. J. Baker , J. A. Carr , et al. 2014. “Mechanisms Underpinning Climatic Impacts on Natural Populations: Altered Species Interactions Are More Important Than Direct Effects.” Global Change Biology 20, no. 7: 2221–2229. 10.1111/gcb.12559. 24677405

[gcb70158-bib-0075] Oudman, T. , K. Laland , G. Ruxton , I. Tombre , P. Shimmings , and J. Prop . 2020. “Young Birds Switch but Old Birds Lead: How Barnacle Geese Adjust Migratory Habits to Environmental Change.” Frontiers in Ecology and Evolution 7: 502. 10.3389/fevo.2019.00502.

[gcb70158-bib-0076] Ouwehand, J. , A. A. Asso , B. Johnston , et al. 2023. “Experimental Food Supplementation at African Wintering Sites Allows for Earlier and Faster Fuelling and Reveals Large Flexibility in Spring Migration Departure in Pied Flycatchers.” Ardea 111, no. 1 : 343–370. 10.5253/arde.2022.a37.

[gcb70158-bib-0077] Pokrovsky, I. , T. Curk , A. Dietz , et al. 2024. “Foxtrot Migration and Dynamic Over‐Wintering Range of an Arctic Raptor.” eLife 12: RP87668. 10.7554/eLife.87668.4. 39513683 PMC11548880

[gcb70158-bib-0078] Pokrovsky, I. , A. Kölzsch , S. Sherub , et al. 2021. “Longer Days Enable Higher Diurnal Activity for Migratory Birds.” Journal of Animal Ecology 90, no. 9: 2161–2171. 10.1111/1365-2656.13484. 33759198

[gcb70158-bib-0079] Post, E. , B. A. Steinman , and M. E. Mann . 2018. “Acceleration of Phenological Advance and Warming With Latitude Over the Past Century.” Scientific Reports 8, no. 1: 1–8. 10.1038/s41598-018-22258-0. 29500377 PMC5834618

[gcb70158-bib-0080] Prop, J. , and J. de Vries . 1993. “Impact of Snow and Food Conditions on the Reproductive Performance of Barnacle Geese *Branta leucopsis* .” Ornis Scandinavia 24, no. 2: 110–121.

[gcb70158-bib-0081] Rakhimberdiev, E. , S. Duijns , C. J. Camphuysen , et al. 2018. “Fuelling Conditions at Staging Sites Can Mitigate Arctic Warming Effects in a Migratory Bird.” Nature Communications 9: 4263. 10.1038/s41467-018-06673-5. PMC6189115 30323300

[gcb70158-bib-0082] Rantanen, M. , A. Karpechko , A. Lipponen , et al. 2022. “The Arctic Has Warmed Nearly Four Times Faster Than the Globe Since 1979.” Communications Earth & Environment 3, no. 1: 168. 10.1038/s43247-022-00498-3.

[gcb70158-bib-0083] Rees, E. 2010. Bewick's Swan. A&C Black.

[gcb70158-bib-0084] Reneerkens, J. , N. M. Schmidt , O. Gilg , et al. 2016. “Effects of Food Abundance and Early Clutch Predation on Reproductive Timing in a High Arctic Shorebird Exposed to Advancements in Arthropod Abundance.” Ecology and Evolution 6, no. 20: 7375–7386. 10.1002/ece3.2361. 28725405 PMC5513252

[gcb70158-bib-0085] Reneerkens, J. , T. S. L. Versluijs , T. Piersma , et al. 2019. “Low fitness at low latitudes: Wintering in the tropics increases migratory delays and mortality rates in an Arctic breeding shorebird.” Journal of Animal Ecology 89, no. 3: 691–703. 10.1111/1365-2656.13118. 31584198 PMC7078868

[gcb70158-bib-0086] Schmidt, N. M. , T. Kankaanpää , M. Tiusanen , et al. 2023. “Little Directional Change in the Timing of Arctic Spring Phenology Over the Past 25 Years.” Current Biology 33, no. 15: 3244–3249.e3. 10.1016/j.cub.2023.06.038. 37499666

[gcb70158-bib-0087] Schreven, K. H. T. , C. Stolz , J. Madsen , and B. A. Nolet . 2021. “Nesting Attempts and Success of Arctic‐Breeding Geese Can Be Derived With High Precision From Accelerometry and GPS‐Tracking.” Animal Biotelemetry 9, no. 1: 1–13. 10.1186/s40317-021-00249-9.

[gcb70158-bib-0088] Semenchuk, P. R. , M. A. K. Gillespie , S. B. Rumpf , N. Baggesen , B. Elberling , and E. J. Cooper . 2016. “High Arctic Plant Phenology Is Determined by Snowmelt Patterns but Duration of Phenological Periods Is Fixed: An Example of Periodicity.” Environmental Research Letters 11, no. 12: 125006. 10.1088/1748-9326/11/12/125006.

[gcb70158-bib-0089] Sergio, F. , A. Tanferna , R. De Stephanis , et al. 2014. “Individual Improvements and Selective Mortality Shape Lifelong Migratory Performance.” Nature 515, no. 7527: 410–413. 25252973 10.1038/nature13696

[gcb70158-bib-0090] Shaftel, R. , D. J. Rinella , E. Kwon , et al. 2021. “Predictors of Invertebrate Biomass and Rate of Advancement of Invertebrate Phenology Across Eight Sites in the North American Arctic.” Polar Biology 44, no. 2: 0123456789. 10.1007/s00300-020-02781-5.

[gcb70158-bib-0091] Smith, P. A. , H. G. Gilchrist , M. R. Forbes , J. L. Martin , and K. Allard . 2010. “Inter‐Annual Variation in the Breeding Chronology of Arctic Shorebirds: Effects of Weather, Snow Melt and Predators.” Journal of Avian Biology 41, no. 3: 292–304. 10.1111/j.1600-048X.2009.04815.x.

[gcb70158-bib-0092] Tavera, E. A. , D. B. Lank , D. C. Douglas , et al. 2024. “Why Do Avian Responses to Change in Arctic Green‐Up Vary?” Global Change Biology 30, no. 5: e17335. 10.1111/gcb.17335. 38771086

[gcb70158-bib-0093] Thackeray, S. J. , P. A. Henrys , D. Hemming , et al. 2016. “Phenological Sensitivity to Climate Across Taxa and Trophic Levels.” Nature 535, no. 7611: 241–245. 10.1038/nature18608. 27362222

[gcb70158-bib-0094] Thackeray, S. J. , T. H. Sparks , M. Frederiksen , et al. 2010. “Trophic Level Asynchrony in Rates of Phenological Change for Marine, Freshwater and Terrestrial Environments.” Global Change Biology 16, no. 12: 3304–3313. 10.1111/j.1365-2486.2010.02165.x.

[gcb70158-bib-0095] Tombre, I. M. , K. A. Høgda , J. Madsen , et al. 2008. “The Onset of Spring and Timing of Migration in Two Arctic Nesting Goose Populations: The Pink‐Footed Goose Anser Bachyrhynchus and the Barnacle Goose *Branta leucopsis* .” Journal of Avian Biology 39, no. 6: 691–703. 10.1111/j.1600-048X.2008.04440.x.

[gcb70158-bib-0096] Tombre, I. M. , T. Oudman , P. Shimmings , and L. Griffin . 2019. “Northward Range Expansion in Spring ‐ Staging Barnacle Geese Is a Response to Climate Change and Population Growth, Mediated by Individual Experience.” Global Change Biology 25, no. 11: 3680–3693. 10.1111/gcb.14793. 31475774

[gcb70158-bib-0097] Tulp, I. , and H. Schekkerman . 2008. “Has Prey Availability for Arctic Birds Advanced With Climate Change? Hindcasting the Abundance of Tundra Arthropods Using Weather and Seasonal Variation.” Arctic 61, no. 1: 48–60. 10.14430/arctic6.

[gcb70158-bib-0098] van Bemmelen, R. S. A. , Y. Kolbeinsson , R. Ramos , et al. 2019. “A Migratory Divide Among Red‐Necked Phalaropes in the Western Palearctic Reveals Contrasting Migration and Wintering Movement Strategies.” Frontiers in Ecology and Evolution 7, no. APR: 1–17. 10.3389/fevo.2019.00086.

[gcb70158-bib-0099] van Bemmelen, R. S. A. , B. Moe , H. Schekkerman , et al. 2024. “Synchronous Timing of Return to Breeding Sites in a Long‐Distance Migratory Seabird With Ocean‐Scale Variation in Migration Schedules.” Movement Ecology 12, no. 1: 22. 10.1186/s40462-024-00459-9. 38520007 PMC10960466

[gcb70158-bib-0100] van Bemmelen, R. V. , B. Moe , S. A. Hanssen , et al. 2017. “Flexibility in Otherwise Consistent Non‐breeding Movements of a Long‐Distance Migratory Seabird the Long‐Tailed Skua.” Marine Ecology Progress Series 578: 197–211. 10.3354/meps12010.

[gcb70158-bib-0101] van der Jeugd, H. , K. Osterbeek , B. J. Ens , J. Shamoun‐Baranes , and K. Exo . 2014. “Data From: Forecasting Spring From Afar? Timing of Migration and Predictability of Phenology Along Different Migration Routes of an Avian Herbivore [Barents Sea Data].” Movebank Data Repository. 10.5441/001/1.ps244r11. 25117616

[gcb70158-bib-0102] van der Jeugd, H. P. , G. Eichhorn , K. E. Litvin , et al. 2009. “Keeping Up With Early Springs: Rapid Range Expansion in an Avian Herbivore Incurs a Mismatch Between Reproductive Timing and Food Supply.” Global Change Biology 15, no. 5: 1057–1071. 10.1111/j.1365-2486.2008.01804.x.

[gcb70158-bib-0103] van Wijk, R. E. , A. Kölzsch , H. Kruckenberg , B. S. Ebbinge , G. J. D. M. Müskens , and B. A. Nolet . 2012. “Individually Tracked Geese Follow Peaks of Temperature Acceleration During Spring Migration.” Oikos 121, no. 5: 655–664. 10.1111/j.1600-0706.2011.20083.x.

[gcb70158-bib-0104] Verhoeven, M. A. , A. H. J. Loonstra , A. D. McBride , et al. 2020. “Geolocators Lead to Better Measures of Timing and Renesting in Black‐Tailed Godwits and Reveal the Bias of Traditional Observational Methods.” Journal of Avian Biology 51, no. 4: 1–12. 10.1111/jav.02259.

[gcb70158-bib-0105] Vermote, E. , and R. Wolfe . 2021. MODIS/Terra Surface Reflectance Daily L2G Global 1km and 500m SIN Grid V061 [Dataset]. NASA EOSDIS Land Processes Distributed Active Archive Center. 10.5067/MODIS/MOD09GA.061.

[gcb70158-bib-0106] Versluijs, T. S. L. 2024. “RGEE_Snowmelt (Version v1.1.0) [Computer Software].” *Zenodo*. 10.5281/zenodo.12802717.

[gcb70158-bib-0107] Visser, M. E. , A. C. Perdeck , J. H. van Balen , and C. Both . 2009. “Climate Change Leads to Decreasing Bird Migration Distances.” Global Change Biology 15, no. 8: 1859–1865. 10.1111/j.1365-2486.2009.01865.x.

[gcb70158-bib-0108] Visser, M. E. , L. te Marvelde , and M. E. Lof . 2012. “Adaptive Phenological Mismatches of Birds and Their Food in a Warming World.” Journal of Ornithology 153, no. 1: 75–84. 10.1007/s10336-011-0770-6.

[gcb70158-bib-0109] Volkov, S. V. , and V. I. Pozdnyakov . 2021. “Effects of Environmental Conditions on Spring Arrival, the Timing of Nesting, and the Reproductive Effort of Ross's Gull (*Phodostethia rosea*) in the Delta of Lena River, Yakutia.” Biology Bulletin 48, no. 8: 1332–1341. 10.1134/S1062359021080318.

[gcb70158-bib-0110] Wood, S. 2023. mgcv: Mixed GAM Computation Vehicle with Automatic Smoothness Estimation (p. 1.9‐1) [Computer software]. CRAN. https://CRAN.R‐project.org/package=mgcv.

[gcb70158-bib-0111] Zhemchuzhnikov, M. K. , T. S. L. Versluijs , T. K. Lameris , J. Reneerkens , C. Both , and J. A. van Gils . 2021. “Exploring the Drivers of Variation in Trophic Mismatches: A Systematic Review of Long‐Term Avian Studies.” Ecology and Evolution 11, no. 9: 3710–3725. 10.1002/ece3.7346. 33976770 PMC8093693

[gcb70158-bib-0112] Boelman, N. T. , J. S. Krause , S. K. Sweet , et al. 2017. “Extreme Spring Conditions in the Arctic Delay Spring Phenology of Long‐Distance Migratory Songbirds.” Oecologia 185, no. 1: 69–80. 10.1007/s00442-017-3907-3. 28779226

[gcb70158-bib-0113] Ely, C. R. , B. J. McCaffery , and R. E. Gill Jr. 2018. “Shorebirds Adjust Spring Arrival Schedules With Variable Environmental Conditions: Four Decades of Assessment on the Yukon–Kuskokwim Delta, Alaska.” In Trends and Traditions: Avifaunal Change in Western North America, edited by W. D. Shuford , R. E. Gill Jr. , and C. M. Handel , 296–311. Western Field Ornithologists. 10.21199/SWB3.16.1.

[gcb70158-bib-0114] Hupp, J. W. , D. H. Ward , D. X. Soto , and K. A. Hobson . 2018. “Spring Temperature, Migration Chronology, and Nutrient Allocation to Eggs in Three Species of Arctic‐Nesting Geese: Implications for Resilience to Climate Warming.” Global Change Biology 24, no. 11: 5056–5071. 10.1111/gcb.14418. 30092605

[gcb70158-bib-0115] Lameris, T. K. , H. P. van der Jeugd , G. Eichhorn , et al. 2018. “Arctic Geese Tune Migration to a Warming Climate but Still Suffer From a Phenological Mismatch.” Current Biology 28: 2467–2473. 10.1016/j.cub.2018.05.077. 30033332

[gcb70158-bib-0116] Lenth, R. V. 2017. “emmeans: Estimated Marginal Means, aka Least‐Squares Means (p. 1.10.4).” [Dataset]. 10.32614/CRAN.package.emmeans.

[gcb70158-bib-0117] Lindberg, M. S. , J. S. Sedinger , and P. L. Flint . 1997. “Effects of Spring Environment on Nesting Phenology and Clutch Size of Black Brant.” Condor 99, no. 2: 381–388. 10.2307/1369944.

[gcb70158-bib-0118] Perkins, D. E. , P. A. Smith , and H. G. Gilchrist . 2007. “The Breeding Ecology of Ruddy Turnstones ( *Arenaria interpres* ) in the Eastern Canadian Arctic.” Polar Record 43, no. 2: 135–142.

[gcb70158-bib-0119] Rakhimberdiev, E. , S. Duijns , C. J. Camphuysen , et al. 2018. “Fuelling Conditions at Staging Sites Can Mitigate Arctic Warming Effects in a Migratory Bird.” Nature Communications 9: 4263. 10.1038/s41467-018-06673-5. PMC6189115 30323300

[gcb70158-bib-0120] Ruthrauff, D. R. , V. P. Patil , J. W. Hupp , and D. H. Ward . 2021. “Life‐History Attributes of Arctic‐Breeding Birds Drive Uneven Responses to Environmental Variability Across Different Phases of the Reproductive Cycle.” Ecology and Evolution 11, no. 24: 18514–18530. 10.1002/ece3.8448. 35003689 PMC8717281

[gcb70158-bib-0121] Volkov, S. V. , and V. I. Pozdnyakov . 2021. “Effects of Environmental Conditions on Spring Arrival, the Timing of Nesting, and the Reproductive Effort of Ross's Gull (*Phodostethia rosea*) in the Delta of Lena River, Yakutia.” Biology Bulletin 48, no. 8: 1332–1341. 10.1134/S1062359021080318.

