# Supplementary material for: Migratory Birds Advance Spring Arrival and Egg‐Laying in the Arctic, Mostly by Travelling Faster
Source: Glob Chang Biol. 2025 Apr 9;31(4):e70158. doi: 10.1111/gcb.70158 (PMC11979735; doi:10.1111/gcb.70158)
Supplement: Supplementary file 1 — Data S1. [file GCB-31-e70158-s001.docx]

**Migratory birds advance spring arrival and egg-laying in the Arctic, mostly by travelling faster**

Supplemental materials

*Arrival on breeding grounds*

For GPS tracks we also calculated arrival on the breeding grounds, as the first date within a bounding box of 35 km of the breeding site (defined in Table S1). We analysed how date of arrival changed with date of snowmelt in a linear mixed model including arrival date as dependent variable and date of snowmelt, population and their interaction as independent variables. From these LMMs, population-specific trends were inspected including 95% confidence intervals in the ‘emtrends’ function in the package ‘emmeans’ (Lenth, 2017). Arrival date at the breeding grounds advanced with earlier date of snowmelt (model including date of snowmelt was 3.8 lower in AICc compared to model without, Figure S2) with no difference between populations (model including interaction between date of snowmelt and population was 1.5 higher in AIC compared to model without). Population-specific trends were similar to trends in arrival in the Arctic, with slopes with 95% CI (of the model including the interaction between population and date of snowmelt) not overlapping with 0 for greater white-fronted geese (0.67 ± 0.11 day earlier arrival per earlier day of snowmelt, mean ± sd), tundra swan (0.46 ± 0.08), but in addition also for barnacle geese breeding on Kolguyev Island (0.31 ± 0.10).

*Stopover sites*

For each day between departure and arrival, we defined whether an individual was staging at a stopover site (distance between daily averaged positions was smaller than 100 km for three consecutive days) or travelling. We then calculated, per individual, the relative time spent at stopovers (days at stopover divided by total days travelling between departure and arrival) and number of unique stopovers visited (Table S3).

*Laying dates tundra swans*

While female swans incubate the eggs like in other waterfowl species, male swans also spend time on the nest, making it less straightforward to derive nesting attempts based solely on GPS locations from females. We adapted the method from Schreven et al (Schreven et al., 2021), which combines GPS and accelerometry data, to account for the lower nest attendance of females. We determined nesting attempts for females aged three years or older based on the age at capture. We calculated the Overall Dynamic Body Acceleration (ODBA; following the method of (Schreven et al., 2021)) of tri-axial accelerometer ‘bursts’ (a consecutive array of measurements starting at a specific timestamp) and determined the daily 0.25 quartile ODBA. We also determined time periods where the ODBA was consistently lower than 1 for at least 30 minutes, which we defined as stationary. Using data from stationary periods only, we calculated the daily median deviation of the latitude from its daily mean, as an indication of movement in geographical space per diem. To determine the onset of incubation, we selected the first three consecutive days on which the daily 0.25 quartile ODBA was below 1 g. Within this period, the first date on which the daily median deviation of the latitude from its daily mean was below 0.001 degrees marked the first day of incubation.


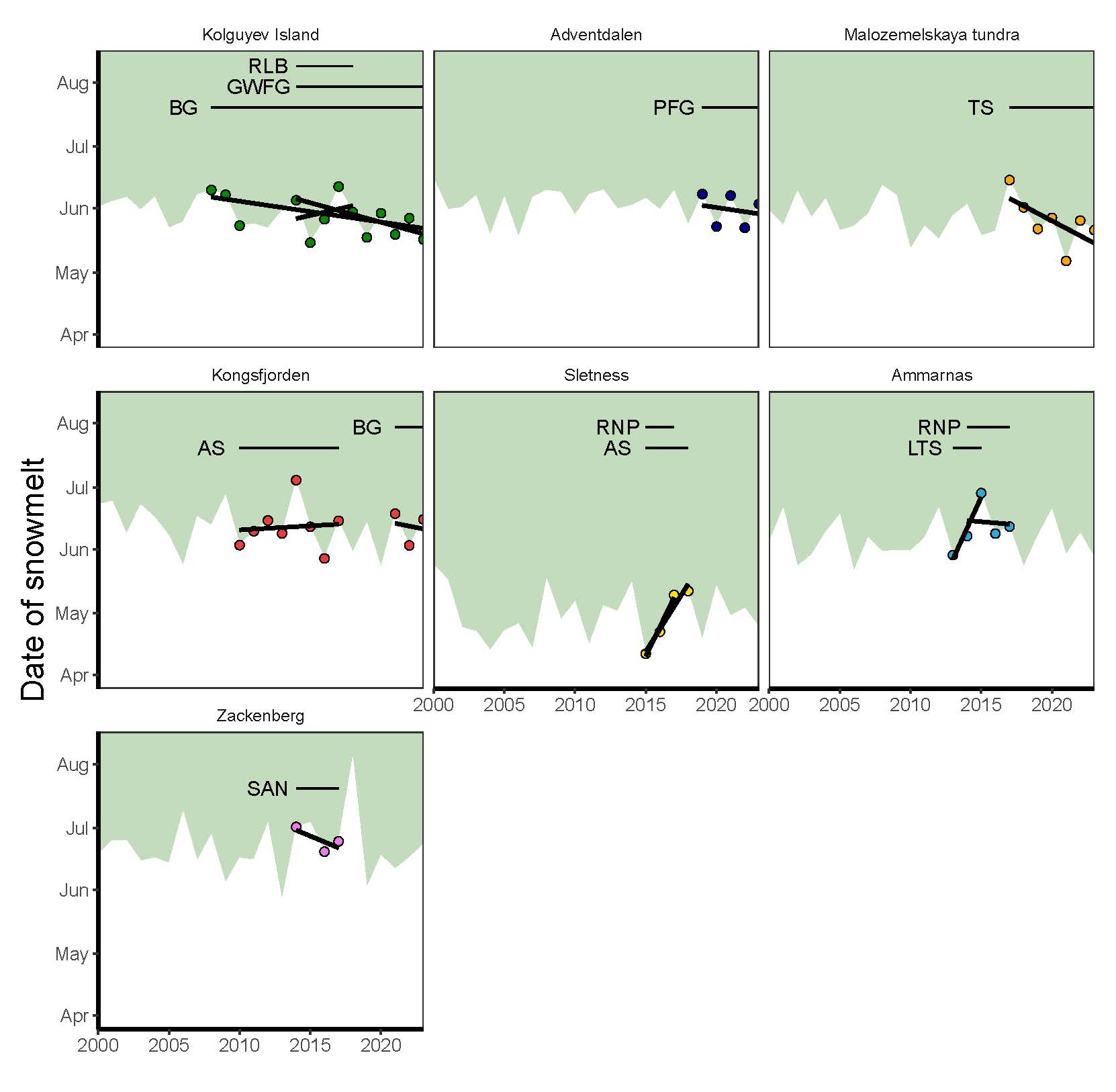


**Figure S1**: Green-white transitions show the date of 50% snowmelt per study sites. Dots show years for which migration and laying data from one or more populations is available, with, horizontal lines in the top of each panel showing the time periods with data for each population (RLB = rough-legged buzzard, GWFG = greater white-fronted goose, BG = barnacle goose, PFG = pink-footed goose, TS = tundra swan, AS = arctic skua, RNP = red-necked phalarope, LTS = long-tailed skua, SAN = sanderling). Thick lines show trends in date of snowmelt for each population.


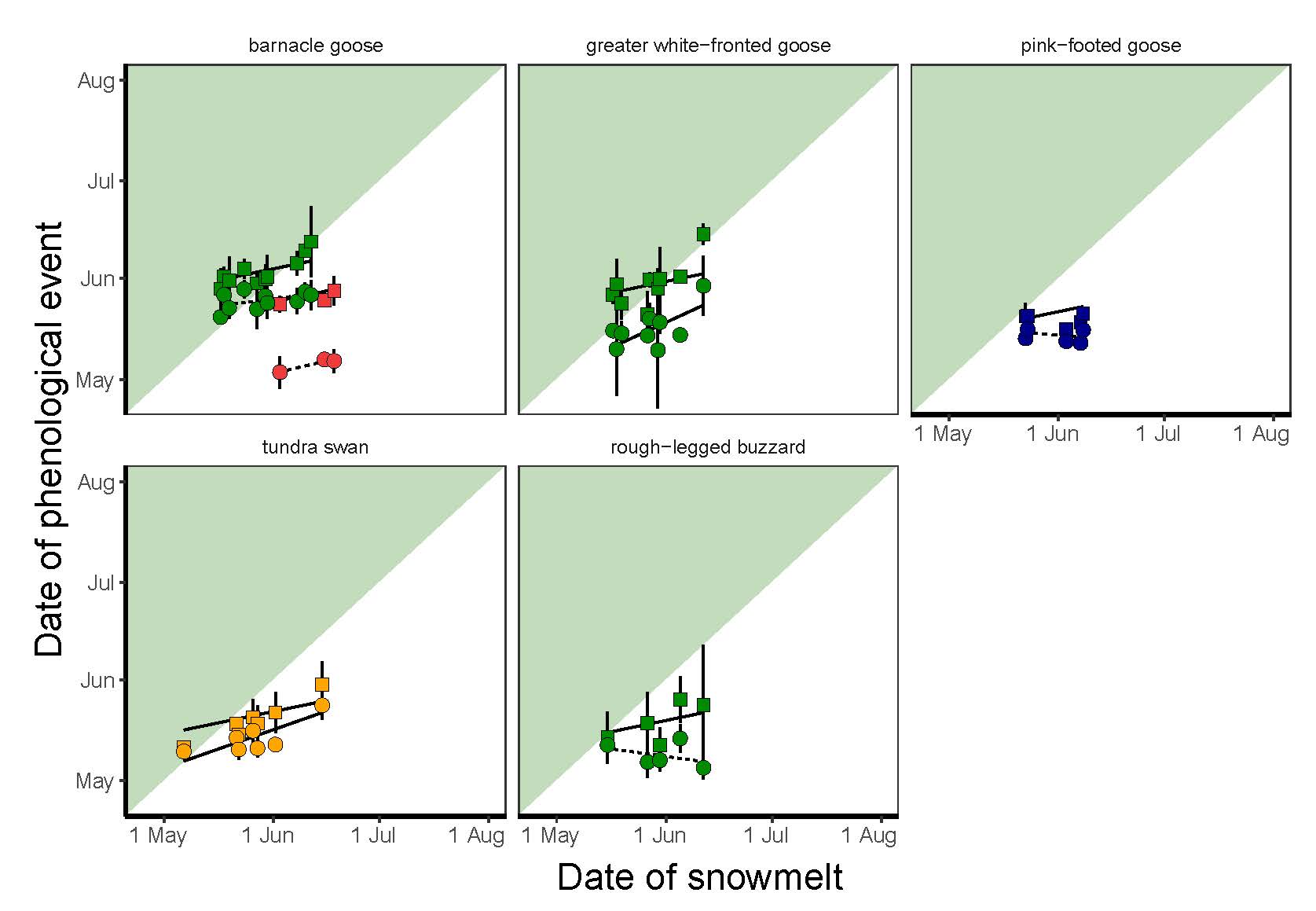


**Figure S2**: Average annual date of arrival on at the Arctic circle (dots) and at breeding sites (squares) for populations tracked with GPS transmitters in relation to the date of 50% snowmelt at the breeding site per species and breeding site. The x=y line segment, where date of arrival or egg-laying equals date of 50% snowmelt, is shown by the white–green transition. Symbols are coloured according to study sites (green = Kolguyev Island, Russia; red = Kongsfjorden, Svalbard, blue = Adventdalen, Svalbard, orange = Malozemelskaya tundra, Russia). Solid lines show trends in date of arrival with day of 50% snowmelt, dashed lines show the mean date of arrival when the trend was not present in models within 2 ΔAICc of the best model. Note that for arrival at the breeding site the best model did not include the interaction between population and date of snowmelt, and all lines show the same slope. Vertical lines display standard deviations.

**Table S1**: Study areas for which snow cover data were extracted from MODIS, including the latitudinal and longitudinal coordinates of each location, number of snow cover pixels retrieved, and

the surface area covered

| **Area** | **Species** | **Latitude** | **Longitude** | **Number of pixels (500m^2^)** | **Surface area (km^2^)** |
| --- | --- | --- | --- | --- | --- |
| Kolguev Island, Russia | barnacle goose, greater white-fronted goose, rough-legged buzzard | 69°05'N | 49°09'E | 24245 | 6061.3 |
| Malozemelskaya Tundra, Russia | tundra swan | 68°22'N | 53°10'E | 60528 | 15132.0 |
| Kongsfjorden, Svalbard, (Blomstrandhalvøya) | Arctic skua, barnacle goose | 78°57'N | 12° 4'E | 32 | 8.0 |
| Adventsdalen, Svalbard | pink-footed goose | 78°10'N | 16°09'E | 225 | 56.3 |
| Ammarnäs, Sweden | long-tailed skua, red-necked phalarope | 66°00'N | 15°53'E | 122 | 30.5 |
| Slettnes, Norway | Arctic skua, red-necked phalarope | 71°05'N | 28°13'E | 40 | 10 |
| Zackenberg, Greenland | sanderling | 74°28'N | 20°34'W | 154 | 19.3 |

* Although both Arctic skuas and barnacle geese breed on islands in the Kongsfjorden, these islands are too small to detect snow cover using the MODIS data set. We therefore chose to use a close by area representing conditions on island in the fjord, namely the southern slope of Blomstrandhalvøya (4 kilometers north of the islands). While this will likely serve as a good proxy, snow may still melt slightly earlier on the islands in Kongsfjordfen.

**Table S2:** Summary of studies from which data were extracted from literature. The methods, how data on timing was acquired (geo = geolocators, GPS = GPS-transmitters, otherwise observed) is provided. The slopes of arrival date at the study site over time (in days per year) and over date of snowmelt (days per later day of snowmelt) are given. Date of snowmelt, i.e. the fraction of a surface area that had melted away, differed between studies.

| Species | Scientific name | Breeding site | Study period | Method spring arrival | Time (years) | | | Date of  snowmelt | | Fraction of snowcover used | Reference |
| --- | --- | --- | --- | --- | --- | --- | --- | --- | --- | --- | --- |
|  |  |  |  |  | spring arrival | lay date | Snow melt | spring arrival | lay date |  |  |
| greater white-fronted goose | Anser  albifrons | Arctic Coastal Plain, Alaska | 2011 - 2013 | tracked (geo) and observed | -1.60 | 0.90 | 2.00 | 0.38 | 1.13 | 50% | Hupp et al., 2018 |
| snow goose | Anser  caerulescens | Arctic Coastal Plain, Alaska | 2011 - 2013 | tracked (geo) and observed | -2.90 | 1.70 | 2.00 | -0.13 | 0.50 | 50% | Hupp et al., 2018 |
| snow goose | Anser  caerulescens | Arctic Coastal Plain, Alaska | 2011 - 2018 | observed |  |  |  | 0.24 | 0.74 | 50% | Ruthrauff et al., 2021 |
| ruddy turnstone | Arenaria  interpres | East Bay, Canada | 2000 - 2003 | observed | 0.10 | -1.70 | -0.10 | 0.39 | 0.43 | 10% | Perkins et al., 2007 |
| black turnstone | Arenaria  melanocephala | Yukon-Kuskokwim delta, Alaska | 1977–1995 | observed | -0.11 |  | 0.17 | 0.31 |  | 10% | Ely et al., 2018 |
| brent goose | Branta  bernicla | Arctic Coastal Plain, Alaska | 2011 - 2018 | observed |  |  |  | 0.24 | 0.84 | 50% | Ruthrauff et al., 2021 |
| brent goose | Branta  bernicla | Yukon-Kuskokwim delta, Alaska | 1987 - 1993 | observed | 0.20 | 0.10 | 0.25 | 0.48 | 0.67 | 0% | Lindberg et al., 1997 |
| brent goose | Branta  bernicla | Arctic Coastal Plain, Alaska | 2011 - 2013 | tracked (geo) and observed | -1.40 | 1.40 | 2.00 | 1.50 | 1.25 | 50% | Hupp et al., 2018 |
| barnacle goose | Branta  leucopsis | West Russian  Arctic | 2003 - 2015 | tracked (GPS) and observed | -0.89 | -0.52 | -1.70 | 0.51 | 0.35 | 50% | Lameris et al., 2018 |
| Lapland longspur | Calcarius lapponicus | Arctic Coastal Plain, Alaska | 2011 - 2018 | observed |  |  |  | -0.08 | 0.38 | 50% | Ruthrauff et al., 2021 |
| Lapland longspur | Calcarius lapponicus | Arctic Coastal Plain, Alaska | 2010 - 2014 | observed | 1.01 | 1.80 | 2.34 | 0.42 | 0.55 | 60% | Boelman et al., 2017 |
| dunlin | Calidris alpina | Yukon-Kuskokwim delta, Alaska | 1977–1995 | observed | 0.00 |  | 0.17 | 0.37 |  | 10% | Ely et al., 2018 |
| western sandpiper | Calidris mauri | Yukon-Kuskokwim delta, Alaska | 1977–1995 | observed | -0.10 |  | 0.17 | 0.41 |  | 10% | Ely et al., 2018 |
| little stint | Calidris minuta | Lena Delta, Russia | 1990 - 2013 | observed | -0.65 | -0.85 | -0.45 | 0.39 | 0.82 | 1% | Volkov, published in Lameris et al. 2025 |
| semipalmated sandpiper | Calidris  pusilla | Arctic Coastal Plain, Alaska | 2011 - 2018 | observed |  |  |  | 0.55 | 0.28 | 50% | Ruthrauff et al., 2021 |
| bar-tailed godwit | Limosa  lapponica | Central-Russian Arctic | 1988 - 2009 | observed | -0.28 | -0.70 | -0.73 | 0.22 | 0.56 | 50% | Rakhimberdiev et al., 2018 |
| bar-tailed godwit | Limosa  lapponica | Yukon-Kuskokwim delta, Alaska | 1977–1995 | observed | -0.04 |  | 0.17 | 0.67 |  | 10% | Ely et al., 2018 |
| red phalarope | Phalaropus fulicarius | Lena Delta, Russia | 1990 - 2013 | observed | -0.17 | -0.18 | -0.45 | 0.40 | 0.42 | 1% | Volkov, published in Lameris et al. 2025 |
| red-necked phalarope | Phalaropus lobatus | Yukon-Kuskokwim delta, Alaska | 1977–1995 | observed | -0.13 |  | 0.17 | 0.28 |  | 10% | Ely et al., 2018 |
| grey plover | Pluvialis squaterola | Yukon-Kuskokwim delta, Alaska | 1977–1995 | observed | -0.14 |  | 0.17 | 0.45 |  | 10% | Ely et al., 2018 |
| Steller's eider | Polysticta stelleri | Lena Delta, Russia | 1990 - 2013 | observed | -0.23 | -0.36 | -0.45 | 0.31 | 0.56 | 1% | Volkov, published in Lameris et al. 2025 |
| Ross' gull | Rhodostethia rosea | Lena Delta, Russia | 1982 - 2013 | observed | -0.44 | -0.42 | -0.45 | 0.59 | 0.78 | 1% | Volkov & Pozdnyakov, 2021 |
| white-crowned sparrow | Zonotrichia lecophrys | Arctic Coastal Plain, Alaska | 2010 - 2014 | observed | -0.34 | 1.50 | 2.34 | 0.05 | 0.36 | 60% | Boelman et al., 2017 |

**Table S3:** LMMs of date of arrival in the Arctic (AC), date of arrival in the breeding area (BA) and laying date (LD) over date of snowmelt (SM), as well as SM, AC and LD over year (Y). Models also included population (P), interactions (e.g. SMxP), and year (Y) and individual identity (ID) as a random intercepts. Models are ordered from smallest to highest ΔAICc. The best performing models are marked in **bold**.

| *Model* | *Degrees of freedom* | *Log-likelyhood* | *AICc* | *Δ AICc* |
| --- | --- | --- | --- | --- |
| **Arrival date over snowmelt** |  |  |  |  |
| **AC ~ SM + P + SMxP + (Y) + (ID)** | **27** | **-1367.39** | **2792.5** | **0.0** |
| AC ~ P + (Y) + (ID) | 15 | -1394.07 | 2819.3 | 26.8 |
| AC ~ SM + P + (Y) + (ID) | 16 | -1394.86 | 2823 | 30.5 |
| AC ~ (Y) + (ID) | 4 | -1552.41 | 3112.9 | 320.4 |
| AC ~ SM +(Y) + (ID) | 5 | -1554.21 | 3118.6 | 326.1 |
| **Laying date over snowmelt** |  |  |  |  |
| **LD ~ SM + P + SMxP + (Y) + (ID)** | **27** | **-1735.42** | **3527.6** | **0.0** |
| LD ~ SM + P + (Y) + (ID) | 16 | -1760.02 | 3553 | 25.4 |
| LD ~ P + (Y) + (ID) | 15 | -1789.89 | 3610.6 | 83.0 |
| LD ~ (Y) + (ID) | 4 | -2262.96 | 4534 | 1006.4 |
| LD ~ SM + (Y) + (ID) | 5 | -2263.26 | 4536.6 | 1009.0 |
| **Breeding arrival over snowmelt** |  |  |  |  |
| **BA ~ SM + P + (Y) + (ID)** | **10** | **-730.23** | **1481.4** | **0.0** |
| BA ~ SM + P + SMxP + (Y) + (ID) | 15 | -725.37 | 1482.9 | 1.5 |
| BA ~ P + (Y) + (ID) | 9 | -733.22 | 1485.2 | 3.8 |
| BA ~ (Y) + (ID) | 4 | -787.45 | 1583.1 | 101.6 |
| BA ~ SM + (Y) + (ID) | 5 | -787.17 | 1584.6 | 103.2 |
| **Snowmelt date over time** |  |  |  |  |
| **SM ~ Y + (ID)** | **3** | **-234.31** | **475.0** | **0.0** |
| **SM ~ (ID)** | **2** | **-236.12** | **476.4** | **1.4** |
| SM ~ Y + P + (ID) | 14 | -232.98 | 502.5 | 27.5 |
| SM ~ P + (ID) | 13 | -234.96 | 503.2 | 28.2 |
| SM ~ Y + P + YxP + (ID) | 25 | -217.47 | 519.1 | 44.1 |
| **Arrival date with time** |  |  |  |  |
| **AC ~ Y + P + (ID)** | **9** | **-902.62** | **1823.9** | **0.0** |
| AC ~ Y + P + YxP + (ID) | 14 | -898.53 | 1826.6 | 2.7 |
| AC ~ P + (ID) | 8 | -909.17 | 1834.9 | 11.0 |
| AC ~ Y + (ID) | 4 | -986.20 | 1980.5 | 156.7 |
| AC ~ (ID) | 3 | -990.98 | 1988 | 164.1 |
| **Laying date with time** |  |  |  |  |
| **LD ~ Y + P + YxP + (ID)** | **14** | **-589.70** | **1209.7** | **0.0** |
| LD ~ Y + P + (ID) | 9 | -600.07 | 1219.1 | 9.4 |
| LD ~ P + (ID) | 8 | -606.70 | 1230.2 | 20.5 |
| LD ~ Y + (ID) | 4 | -648.34 | 1304.9 | 95.2 |
| LD ~ (ID) | 3 | -653.88 | 1313.9 | 104.2 |

**Table S4:** LMs of slopes of migratory arrival over time (SwT) and over date of snowmelt (SwSM) as dependent variable, and slopes of travel speed (TS), departure date (Dp) and migration distance (Ds) over time / snowmelt as independent variables. Models are ordered from smallest to highest ΔAICc. The best performing model is marked in **bold**.

| *Model* | *Degrees of freedom* | *Log-likelyhood* | *AICc* | *Δ AICc* |
| --- | --- | --- | --- | --- |
| **Slopes with date of snowmelt** |  |  |  |  |
| **SwSM ~ 1** | **2** | **20.011** | **-34.7** | **0** |
| SwSM ~ TS | 3 | 21.532 | -34.1 | 0.62 |
| SwSM ~ Dp | 4 | 23.888 | -34.1 | 0.63 |
| SwSM ~ Ds | 3 | 20.913 | -32.8 | 1.86 |
| SwSM ~ Dp + TS | 3 | 20.54 | -32.1 | 2.61 |
| SwSM ~ Ds + TS | 4 | 21.995 | -30.3 | 4.41 |
| SwSM ~ Dp + Ds | 4 | 20.978 | -28.2 | 6.45 |
| SwSM ~ Dp + Ds + TS | 5 | 23.971 | -27.9 | 6.75 |
| **Slopes with time** |  |  |  |  |
| **SwT ~ TS** | **3** | **10.386** | **-2.8** | **0** |
| SwT ~ 1 | 2 | 2.964 | 2.1 | 4.84 |
| SwT ~ Dr | 3 | 3.19 | 11.6 | 14.39 |
| SwT ~ Ds | 3 | 2.965 | 12.1 | 14.84 |
| SwT ~ Ds + TS | 4 | 10.99 | 26 | 28.79 |
| SwT ~ Dp + TS | 4 | 10.402 | 27.2 | 29.97 |
| SwT ~ Dp + Ds | 4 | 3.617 | 40.8 | 43.54 |
| SwT ~ Dp + Ds + TS | 5 | 13.226 | Infinite | Infinite |

**Table S5:** LMMs of lay date (LD) over date of arrival in the Arctic (AC), date of snowmelt (SM), population (P) including interactions (e.g. SMxP), and year (Y) and individual identity (ID) as a random intercepts. Models are ordered from smallest to highest ΔAICc. The best performing model is marked in **bold**.

| *Model* | *Degrees of freedom* | *Log-likelyhood* | *AICc* | *Δ AICc* |
| --- | --- | --- | --- | --- |
| **LD ~ AC + SM + P + (Y) + (ID)** | **13** | **-943.59** | **1914.4** | **0** |
| LD ~ AC + SM + (Y) + (ID) | 6 | -953.787 | 1919.8 | 5.45 |
| LD ~ AC + SM + P + ACxP + (Y) + (ID) | 20 | -939.038 | 1921 | 6.58 |
| LD ~ SM + P + (Y) + (ID) | 12 | -948.957 | 1923 | 8.57 |
| LD ~ AC + SM + P + SMxP + (Y) + (ID) | 20 | -940.647 | 1924.2 | 9.79 |
| LD ~ SM + P + SMxP + (Y) + (ID) | 19 | -942.861 | 1926.3 | 11.94 |
| LD ~ AC + P + (Y) + (ID) | 12 | -952.237 | 1929.5 | 15.13 |
| LD ~ SM + (Y) + (ID) | 5 | -960.227 | 1930.7 | 16.25 |
| LD ~ AC + SM + P + ACxP + SMxP + (Y) + (ID) | 27 | -936.687 | 1932.7 | 18.32 |
| LD ~ AC + (Y) + (ID) | 5 | -963.201 | 1936.6 | 22.2 |
| LD ~ AC + P + ACxP + (Y) + (ID) | 19 | -948.511 | 1937.6 | 23.24 |
| LD ~ P + (Y) + (ID) | 11 | -957.77 | 1938.4 | 24.03 |
| LD ~ (Y) + (ID) | 4 | -970.153 | 1948.4 | 34.04 |

**Table S6:** Number of stopover sites and the fraction of time spent at stopovers (days at stopover divided by total days travelling between departure and arrival) below the Arctic circle. Means ± standard deviations are presented for each population.

| Species | Study site  (breeding location) | Number of stopover sites | Fraction of time at stopover sites |
| --- | --- | --- | --- |
| *barnacle goose* | Kolguev Island, Russia | 1.0 ± 1.2 | 0.36 ± 0.36 |
|  | Kongsfjorden, Svalbard | 0.1 ± 0.3* | 0.08 ± 0.24 |
| *greater white-fronted goose* | Kolguev Island, Russia | 3.5 ± 1.7 | 0.85 ± 0.07 |
| *pink-footed goose* | Adventdalen, Svalbard | 1.2 ± 0.5 | 0.92 ± 0.01 |
| *tundra swan* | Malozemelskaya tundra, Russia | 3.4 ± 1.3 | 0.82 ± 0.05 |
| *red-necked phalarope* | Ammarnäs, Sweden | 1.5 ± 0.7 | 0.44 ± 0.19 |
|  | Slettnes, Norway | 1.6 ± 0.8 | 0.54 ± 0.22 |
| *sanderling* | Zackenberg, Greenland | 2.1 ± 0.6 | 0.69 ± 0.09 |
| *Arctic skua* | Kongsfjorden, Svalbard | 0.1 ± 0.4 | 0.02 ± 0.07 |
|  | Slettnes, Norway | 0.5 ± 0.7 | 0.08 ± 0.11 |
| *long-tailed skua* | Ammarnäs, Sweden | 1.3 ± 1.4 | 0.08 ± 0.08 |
| *rough-legged buzzard* | Kolguev Island, Russia | 1.0 ± 1.2 | 0.30 ± 0.31 |

* The low number of stopover sites for this population is because most individuals stopover in northern Norway just above the Arctic circle (Tombre et al., 2019). This did not affect trends in arrival with date of snowmelt, as also arrival in the breeding area was unrelated to date of snowmelt.

**References**

Boelman, N. T., Krause, J. S., Sweet, S. K., Chmura, H. E., Perez, J. H., Gough, L., & Wingfield, J. C. (2017). Extreme spring conditions in the Arctic delay spring phenology of long-distance migratory songbirds. *Oecologia*, *185*(1), 69–80. https://doi.org/10.1007/s00442-017-3907-3

Ely, C. R., McCaffery, B. J., & Gill, R. E. Jr. (2018). Shorebirds adjust spring arrival schedules with variable environmental conditions: Four decades of assessment on the Yukon–Kuskokwim Delta, Alaska. In W. D. Shuford, G. Jr. R. E., & C. M. Handel (Eds.), *Trends and traditions: Avifaunal change in western North America* (pp. 296–311). Western Field Ornithologists. https://doi.org/10.21199/SWB3.16.1

Hupp, J. W., Ward, D. H., Soto, D. X., & Hobson, K. A. (2018). Spring temperature, migration chronology, and nutrient allocation to eggs in three species of arctic-nesting geese: Implications for resilience to climate warming. *Global Change Biology*, *24*(11), 5056–5071. https://doi.org/10.1111/gcb.14418

Lameris, T.K., Boom, M.P., Nuijten, R.J.M., Buitendijk, N.H., Eichhorn, G., Ens, B.J., Exo, K-M., Glazov, P.M., Are Hanssen, S., Hunke, P., van der Jeugd, H.P., de Jong, M.E., Kölzsch, A., Kondraytev, A., Kruckenberg, H., Kulikova, O., Linssen, H., Loonen, M.J.J.E., Loshchagina, J.A., Madsen, J., Moe, B., Moonen, S., Müskens, G.J.D.M., Nolet, B.A., Pokrovsky, I., Reneerkens, J., Scheiber, I.B.R., Schekkerman, H., Schreven, K.H.T., Tal, T., Tulp, I., Verhoeven, M.A., Versluijs, T.L., Volkov, S., Wikelsi, M., van Bemmelen, R.S.A. (2025). Data from: Migratory birds advance spring arrival and egg-laying in the Arctic, mostly by travelling faster. Dryad Digital Repository. https://doi.org/10.5061/dryad.w0vt4b93d

Lameris, T. K., van der Jeugd, H. P., Eichhorn, G., Dokter, A. M., Bouten, W., Boom, M. P., Litvin, K. E., Ens, B. J., & Nolet, B. A. (2018). Arctic Geese Tune Migration to a Warming Climate but Still Suffer from a Phenological Mismatch. *Current Biology*, *28*, 2467–2473. https://doi.org/10.1016/j.cub.2018.05.077

Lenth, R. V. (2017). *emmeans: Estimated Marginal Means, aka Least-Squares Means* (p. 1.10.4) [Dataset]. https://doi.org/10.32614/CRAN.package.emmeans

Lindberg, M. S., Sedinger, J. S., & Flint, P. L. (1997). Effects of spring environment on nesting phenology and clutch size of Black Brant. *Condor*, *99*(2), 381–388. https://doi.org/10.2307/1369944

Perkins, D. E., Smith, P. A., & Gilchrist, H. G. (2007). The breeding ecology of ruddy turnstones (Arenaria interpres) in the eastern Canadian Arctic. *Polar Record*, *43*(2), 135–142.

Rakhimberdiev, E., Duijns, S., Camphuysen, C. J., Castricum, V., Dekinga, A., Dekker, R., Gavrilov, A., ten Horn, J., Jukema, J., Karagicheva, J., Saveliev, A., Soloviec, M., Tibbitts, T. L., van Gils, J. A., & Piersma, T. (2018). Fuelling conditions at staging sites can mitigate Arctic warming effects in a migratory bird. *Nature Communications*, *9*, 4263. https://doi.org/10.1038/s41467-018-06673-5

Ruthrauff, D. R., Patil, V. P., Hupp, J. W., & Ward, D. H. (2021). Life‐history attributes of Arctic‐breeding birds drive uneven responses to environmental variability across different phases of the reproductive cycle. *Ecology and Evolution*, *11*(24), 18514–18530. https://doi.org/10.1002/ece3.8448

Tombre, I. M., Oudman, T., Shimmings, P., & Griffin, L. (2019). Northward range expansion in spring ‐ staging barnacle geese is a response to climate change and population growth , mediated by individual experience. *Global Change Biology 25*(11), 3680-3693. https://doi.org/10.1111/gcb.14793

Volkov, S. V., & Pozdnyakov, V. I. (2021). Effects of Environmental Conditions on Spring Arrival, the Timing of Nesting, and the Reproductive Effort of Ross’s Gull (*Phodostethia rosea*) in the Delta of Lena River, Yakutia. *Biology Bulletin*, *48*(8), 1332–1341. https://doi.org/10.1134/S1062359021080318
